# Macrophage JAK2 deficiency protects against high-fat diet-induced inflammation

**DOI:** 10.1038/s41598-017-07923-0

**Published:** 2017-08-09

**Authors:** Harsh R. Desai, Tharini Sivasubramaniyam, Xavier S. Revelo, Stephanie A. Schroer, Cynthia T. Luk, Prashanth R. Rikkala, Adam H. Metherel, David W. Dodington, Yoo Jin Park, Min Jeong Kim, Joshua A. Rapps, Rickvinder Besla, Clinton S. Robbins, Kay-Uwe Wagner, Richard P. Bazinet, Daniel A. Winer, Minna Woo

**Affiliations:** 1 Toronto General Hospital Research Institute, University Health Network, Toronto, M5G 1L7 Canada; 2 Department of Laboratory Medicine and Pathobiology, University of Toronto, Toronto, M5S 1A8 Canada; 3 Department of Nutritional Sciences, University of Toronto, Toronto, M5S 3E2 Canada; 4 Institute of Medical Research, Kangbuk Samsung Hospital, Sungkyunkwan University School of Medicine, Seoul, 03181 Korea; 5 Peter Munk Cardiac Centre, University Health Network, Toronto, M5G 1L7 Canada; 6 Eppley Institute for Research in Cancer and Allied Diseases and the Department of Biochemistry and Molecular Biology, University of Nebraska Medical Center, Omaha, 68198 USA; 7 Department of Pathology, University Health Network, Toronto, M5G 2C4 Canada; 8 Division of Endocrinology and Metabolism, Department of Medicine, University Health Network and University of Toronto, Toronto, M5G 2C4 Canada

## Abstract

During obesity, macrophages can infiltrate metabolic tissues, and contribute to chronic low-grade inflammation, and mediate insulin resistance and diabetes. Recent studies have elucidated the metabolic role of JAK2, a key mediator downstream of various cytokines and growth factors. Our study addresses the essential role of macrophage JAK2 in the pathogenesis to obesity-associated inflammation and insulin resistance. During high-fat diet (HFD) feeding, macrophage-specific JAK2 knockout (M-JAK2^−/−^) mice gained less body weight compared to wildtype littermate control (M-JAK2^+/+^) mice and were protected from HFD-induced systemic insulin resistance. Histological analysis revealed smaller adipocytes and qPCR analysis showed upregulated expression of some adipogenesis markers in visceral adipose tissue (VAT) of HFD-fed M-JAK2^−/−^ mice. There were decreased crown-like structures in VAT along with reduced mRNA expression of some macrophage markers and chemokines in liver and VAT of HFD-fed M-JAK2^−/−^ mice. Peritoneal macrophages from M-JAK2^−/−^ mice and *Jak2* knockdown in macrophage cell line RAW 264.7 also showed lower levels of chemokine expression and reduced phosphorylated STAT3. However, leptin-dependent effects on augmenting chemokine expression in RAW 264.7 cells did not require JAK2. Collectively, our findings show that macrophage JAK2 deficiency improves systemic insulin sensitivity and reduces inflammation in VAT and liver in response to metabolic stress.

## Introduction

Macrophages are present in almost all tissues and serve crucial roles in the development, metabolism and maintenance of homeostasis, as well as play an integral part in mediating inflammation^1^. They serve an adaptive function during infection or tissue injury to resolve acute inflammation. However, macrophages can also contribute substantially to the progression of chronic diseases such as obesity. Obesity is associated with a chronic low-grade inflammatory state and can contribute to the pathogenesis of insulin resistance and type 2 diabetes^2^. There appears to be a vicious cycle connecting inflammation and the pathological process it accompanies. Thus, obesity can lead to inflammation, whereas chronic inflammation can promote obesity-associated diabetes in part by inducing insulin resistance^2^. The role of macrophages in the establishment of the chronic inflammatory state and metabolic dysfunction was first identified in adipose tissue macrophages, which were found to account for a substantial percent of the total cell population within the obese adipose tissue of mice and humans^3, 4^.

Metabolic activation of macrophages is not limited to adipose tissue, but obesity-induced inflammatory changes have also been reported in liver, muscle, hypothalamus and pancreas^5^, affirming the significant role of these cells in the physiological response. Inflammatory cytokines and chemokines released by metabolic tissues such as adipose tissue and liver can activate inflammatory signalling within macrophages. Tissue macrophages can change their polarization status in response to changes in the local environment. They can be broadly classified as classically activated macrophages (M1) and alternatively activated macrophages (M2)^6^. In turn, activated M1 macrophages can trigger the release of pro-inflammatory cytokines and chemokines^6^, which can activate distinct intracellular pathways that promote the development of local and systemic insulin resistance^7^.

A major pathway involved in inflammatory signalling is the Janus kinase (JAK)-signal transducers and activators of transcription, which has been implicated in the development of a number of inflammatory diseases including obesity^8^. In particular, JAK2 plays an integral role in regulating a wide variety of physiological processes including lactation, erythropoiesis, myelopoiesis, thrombopoiesis and inflammation^9^. It is a ubiquitously expressed member of the mammalian JAK family of non-receptor tyrosine kinases, which also includes JAKl, JAK3, and Tyk2. It acts as a downstream mediator of various cytokines and growth factors such as interleukin (IL)-3, IL-6, interferon (IFN)-γ, granulocyte-macrophage colony-stimulating factor (GM-CSF), thrombopoietin, erythropoietin, growth hormone, and leptin^9^. The whole body JAK2 knockout mice are embryonic lethal^10, 11^, therefore, tissue-specific functions of JAK2 have been elucidated with Cre recombinase system. Hepatocyte-specific deletion of JAK2 leads to profound fatty liver and hepatic insulin resistance^12, 13^ with a paradoxical protection against diabetes^12^. On the other hand, JAK2 deficiency in adipocytes promotes obesity and insulin resistance^14–16^. However, the function of macrophage JAK2 in the inflammatory response and insulin resistance caused by diet-induced obesity remains unknown.

In the present work, mice with myeloid-specific deletion of JAK2 were generated using the lysozyme M (LysM)-Cre line to assess its role in mature macrophages in the pathogenesis of inflammation and insulin resistance. During high-fat diet (HFD) feeding, macrophage JAK2 knockout mice displayed improved whole body glucose homeostasis and reduced expression of inflammatory factors in the liver and adipose tissue. These results indicate that macrophage JAK2 promotes the development of systemic insulin resistance and inflammation.

## Results

### Generation of a myeloid specific JAK2-deficient mouse model

**Figure 1:**
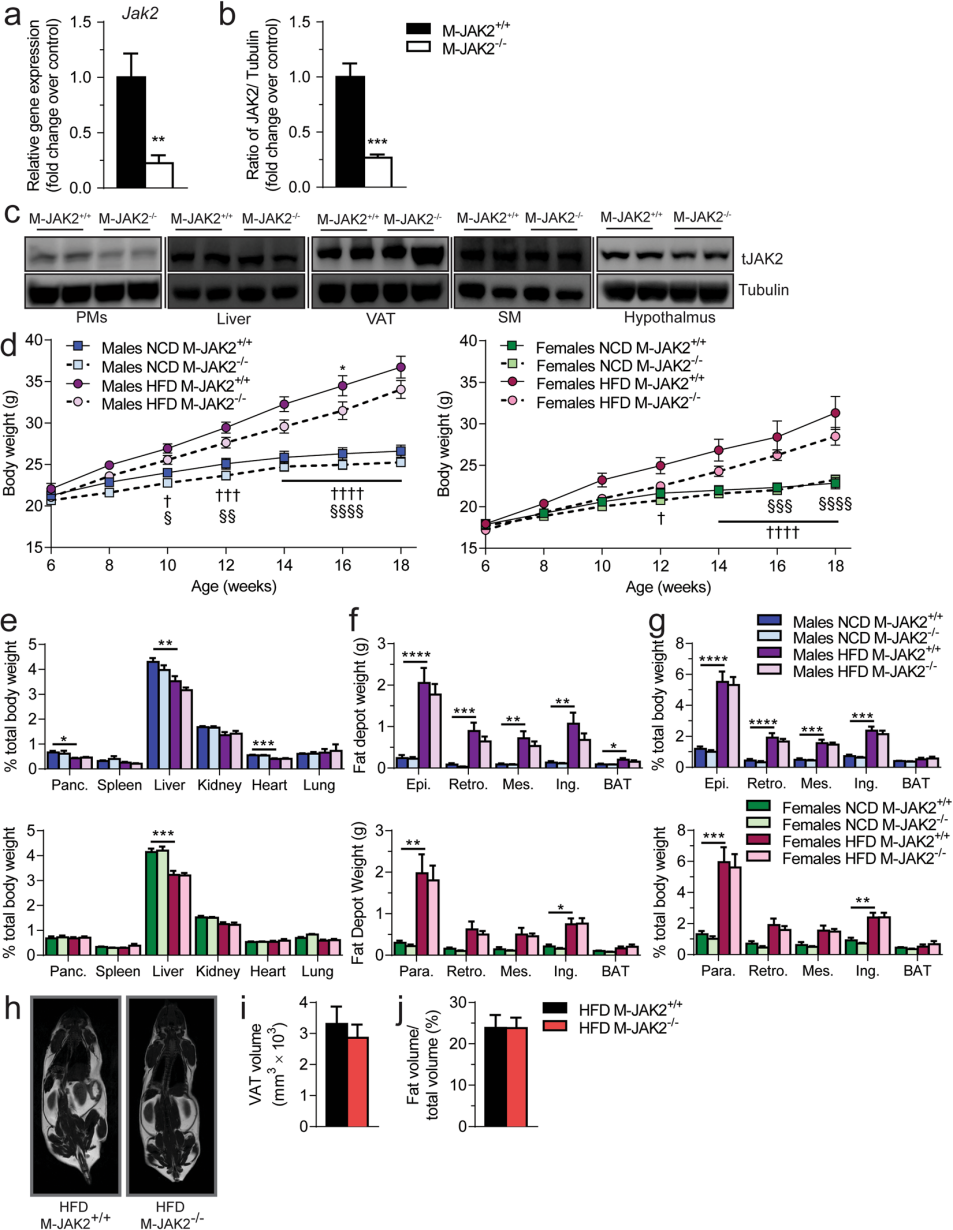
Generation and characterization of M-JAK2^+/+^ and M-JAK2^−/−^ mice. (**a**) mRNA expression and (**b**) protein levels for JAK2 in thioglycolate-elicited peritoneal macrophages (PMs) from 8- to 10-week old M-JAK2^+/+^ and M-JAK2^−/−^ mice (n = 6–7). (**c**) Representative Western blots of protein lysates from PMs, liver, visceral adipose tissue (VAT), skeletal muscle (SM), and hypothalamus from M-JAK2^+/+^ and M-JAK2^−/−^ mice for JAK2 and tubulin, as a loading control. Full blots in Supplementary Fig. S6. (**d**) Body weight measurements of male and female M-JAK2^+/+^ and M-JAK2^−/−^ mice fed NCD (n = 12-13 males, n = 12–13 females) or HFD (n = 13–14 males, n = 11–13 females); *HFD M-JAK2^+/+^ compared to HFD M-JAK2^−/−^ mice, † NCD M-JAK2^+/+^ compared to HFD M-JAK2^+/+^ mice, § NCD M-JAK2^−/−^ compared to HFD M-JAK2^−/−^. (**e**) Tissue weights relative to total body weight of pancreas (Panc.), spleen, liver, kidney, heart, and lungs; and (**f**) absolute weights and (**g**) weights relative to total body weight of adipose depots: epidydimal (epi., male), parametrial (para., female), retroperitoneal (retro.), mesenteric (mes.), inguinal (ing.), and interscapular brown adipose tissue (BAT) in M-JAK2^+/+^ and M-JAK2^−/−^ male and female mice fed NCD (n = 5–8 males, n = 9 females) or HFD (n = 7–9 males, n = 10–11 females). (**h**) Representative image of whole animal MRI scan; (**i**) total VAT volume and (**j**) percentage of abdominal adipose volume relative to total body volume determined using MRI in M-JAK2^+/+^ and M-JAK2^−/−^ male mice fed HFD (n = 4). All results are mean ± SEM; *p < 0.05, **p < 0.01, ***p < 0.001, ****p﻿ < 0.0001.

To investigate the role of signaling by macrophage JAK2 in obesity-related insulin resistance and inflammation *in vivo*, we disrupted *Jak2* by homologous recombination in mice using LysM promoter, which is expressed specifically in cells of the myeloid lineage^17^. To verify the efficiency of *Jak2* deletion, we measured *Jak2* mRNA levels in thioglycolate-elicited peritoneal macrophages (PMs) and observed that they were decreased by approximately 80% in macrophage JAK2 deficient (M-JAK2^−/−^) compared with littermate control mice (M-JAK2^+/+^) (Fig. 1a). Moreover, JAK2 protein expression was reduced by about 75% in M-JAK2^−/−^ PM lysates (Fig. 1b,c) but not in liver, visceral adipose tissue (VAT), skeletal muscle and hypothalamus (Fig. 1c).

### Macrophage JAK2 deficient mice show decreased adipocyte hypertrophy during HFD feeding

To assess the role of macrophage JAK2 in regulating body mass, we fed M-JAK2^−/−^ and M-JAK2^+/+^ mice either a normal chow diet (NCD) or a HFD, and monitored body weight from 6 weeks to 18 weeks of age. Both males and females gained significantly more weight during 12 weeks of HFD feeding than NCD (Fig. 1d). However, a lesser degree of weight gain for male M-JAK2^−/−^ mice was observed transiently compared with M-JAK2^+/+^ mice during HFD feeding (Fig. 1d). These differences in body weight were absent for both male and female M-JAK2^−/−^ and M-JAK2^+/+^ mice fed NCD (Fig. 1d). In addition, when we examined metabolic changes in male M-JAK2^−/−^ compared with that of M-JAK2^+/+^ during NCD or HFD feeding, we did not see any apparent differences in daily food intake, energy expenditure, oxygen consumption, respiratory exchange ratio, or activity level (Supplementary Fig. S1).

**Figure 2:**
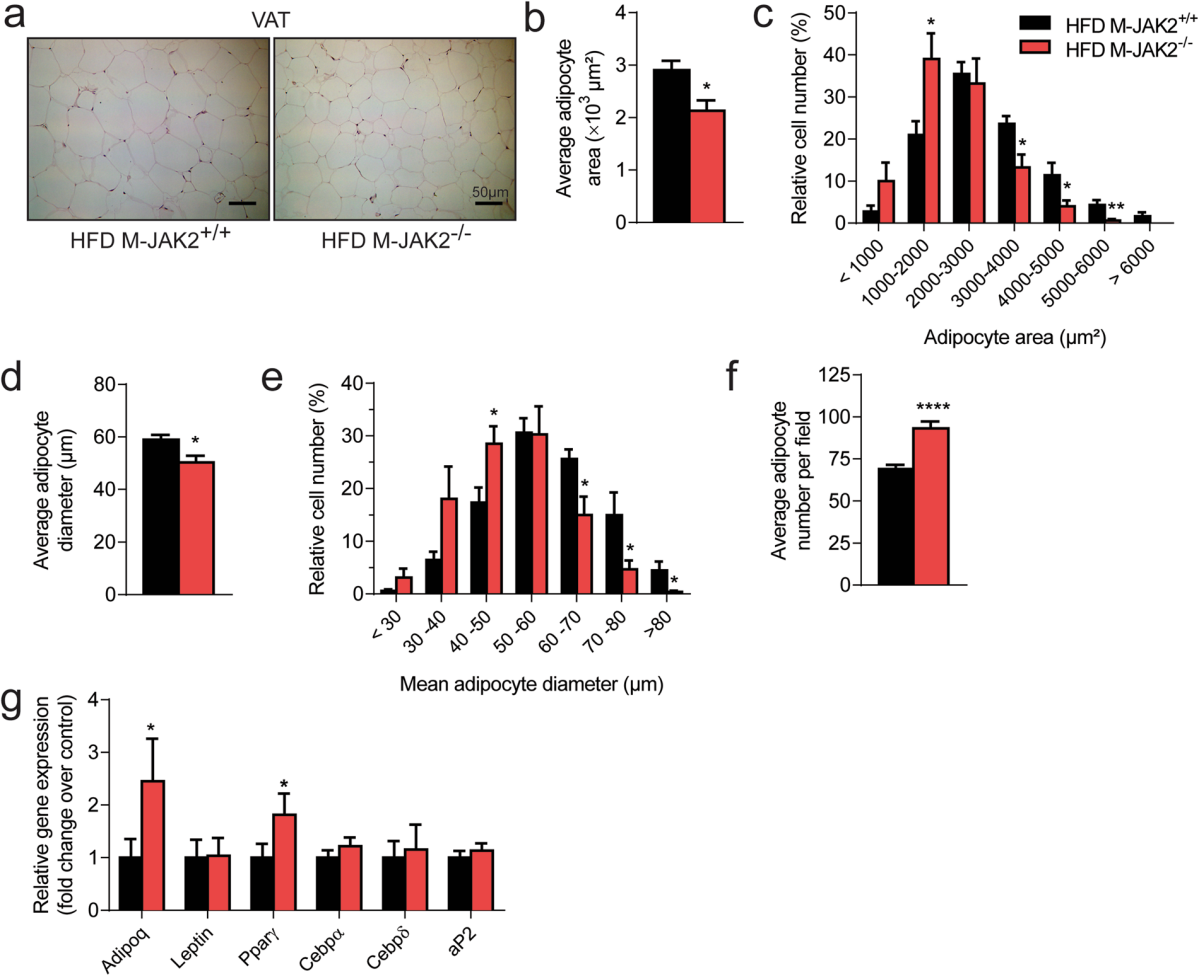
Decreased adipocyte hypertrophy in HFD-fed M-JAK2^−/−^ mice. (**a**) Representative micrographs of H&E stained VAT sections of HFD-fed M-JAK2^+/+^ and M-JAK2^−/−^ mice. Quantitative analysis of adipocytes in VAT sections from HFD fed M-JAK2^+/+^ and M-JAK2^−/−^ (n = 7–9): (**b**) average adipocyte area, (**c**) adipocyte area distribution, (**d**) average adipocyte diameter, (**e**) adipocyte diameter distribution, and (**f**) adipocyte number per field. (**g**) mRNA expression of genes involved in adipogenesis in VAT of HFD fed M-JAK2^+/+^ and M-JAK2^−/−^ mice (n = 8–10). All results are mean ± SEM; *p < 0.05, **p < 0.01, ****p < 0.0001.

We next assessed organ weights at necropsy in both male and female mice fed NCD or HFD. There were no differences in relative weights of all tissues in NCD- or HFD-fed M-JAK2^−/−^ compared to controls (Fig. 1e). We observed increased adiposity in male and female mice with HFD feeding compared to NCD fed mice with no significant difference between genotypes for weights of white adipose depots from NCD or HFD fed male and female M-JAK2^−/−^ mice (Fig. 1f,g). Similarly, we did not see a difference in VAT volume by MRI imaging between male M-JAK2^+/+^ and M-JAK2^−/−^ (Fig. 1h,i). Interestingly, histological analysis of VAT performed on the HFD fed cohort revealed increased proportion of smaller adipocytes in haematoxylin and eosin (H & E) stained VAT sections from M-JAK2^−/−^ mice (Fig. 2a) with reduced mean adipocyte area (Fig. 2b,c) and diameter (Fig. 2d,e) compared to those of M-JAK2^+/+^ littermates. We also saw a significantly increased number of adipocytes per section (Fig. 2f). The differences in adipocyte size led us to examine mRNA expression in VAT for genes involved in adipogenesis, a process for adipocyte turnover or renewal. In support of increased adipogenesis and maturation of adipocytes, we found that M-JAK2^−/−^ mice fed HFD had upregulated expression of *Adipoq* and *Ppar*γ (Fig. 2g). Our results reveal that during HFD feeding, VAT from macrophage JAK2 deficient mice displayed decreased adipocyte hypertrophy and enhancement of some adipogenic gene expression compared to control littermates.

### Lack of macrophage JAK2 results in protection from HFD induced systemic insulin resistance

**Figure 3:**
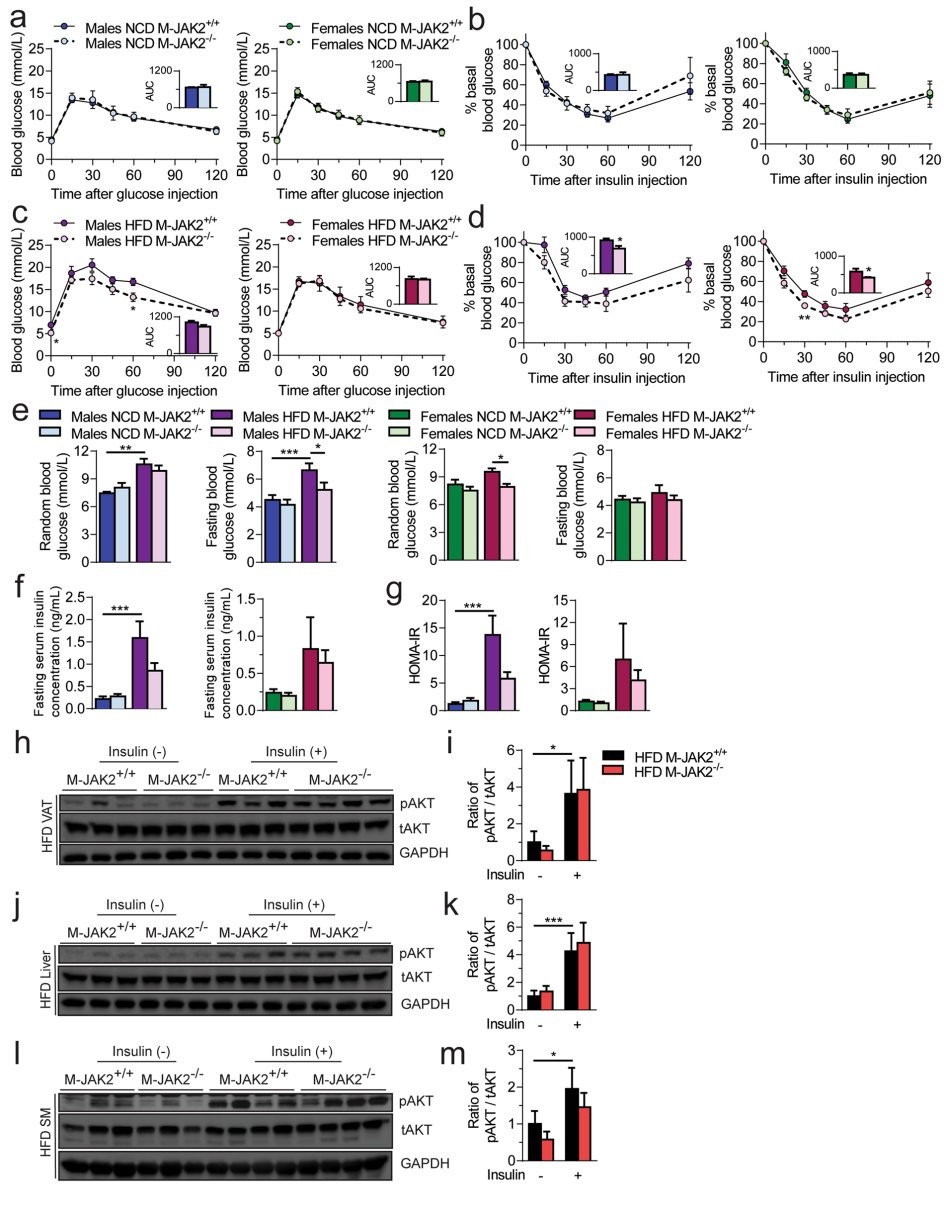
Increased systemic insulin sensitivity in HFD fed M-JAK2^−/−^ mice. Male and female M-JAK2^+/+^ and M-JAK2^−/−^ mice subjected to glucose tolerance test during (**a**) NCD feeding (n = 5–8 males, n = 9 females) or (**b**) HFD feeding (n = 9–10 males, n = 11–13 females) feeding. Male and female M-JAK2^+/+^ and M-JAK2^−/−^ mice subjected to insulin tolerance test during (**c**) NCD feeding (n = 5–8 males, n = 8 females) or (**d**) HFD feeding (n = 7–9 males, n = 9–12 females) feeding. Area under the curve (AUC) was measured using baseline glucose values. (**e**) Random and fasting blood glucose levels measured in male and female M-JAK2^+/+^ and M-JAK2^−/−^ mice during NCD feeding (n = 5–8 males, n = 9 females) or HFD feeding (n = 9–10 males, n = 9–12 females). (**f**) Fasting serum insulin levels and (**g**) homeostasis model assessment of insulin resistance (HOMA-IR) calculated in male and female M-JAK2^+/+^ and M-JAK2^−/−^ mice fed NCD (n = 6–7 males, n = 7–8 females) or HFD (n = 9–10 males, n = 8–10 females). Western blots of protein lysates from (**h**) visceral adipose tissue (VAT), (**j**) liver and (**l**) skeletal muscle (SM) for phospho (Ser473)-AKT (pAKT), total AKT (tAKT) and GAPDH as a loading control, and quantification of protein levels expressed as fold change over M-JAK2^+/+^ injected without insulin in (**i**) VAT, (**k**) liver and (**m**) SM from HFD fed M-JAK2^+/+^ and M-JAK2^−/−^ mice injected without (−) or with ( + ) insulin (n = 3–4). Full blots in Supplementary Fig. S7. All results are mean ± SEM; *p < 0.05, **p < 0.01, ***p < 0.001.

Next, we investigated alterations in glucose homeostasis after prolonged HFD feeding. At 18 weeks of age, we subjected M-JAK2^−/−^ and M-JAK2^+/+^ mice to intraperitoneal (i.p.) glucose tolerance test (GTT) and i.p. insulin tolerance test (ITT). Male and female M-JAK2^−/−^ mice and M-JAK2^+/+^ control cohort fed NCD had no significant difference in the glucose profile during the GTT and ITT (Fig. 3a,b). Interestingly, HFD-fed male and female M-JAK2^−/−^ mice showed comparable glucose tolerance (Fig. 3c), but enhanced glucose lowering during insulin tolerance test than M-JAK2^+/+^ control mice (Fig. 3d). M-JAK2^−/−^ and M-JAK2^+/+^ mice fed NCD showed no genotype-related difference in fasting and random blood glucose levels (Fig. 3e). However, HFD-fed male M-JAK2^−/−^ exhibited reduced fasting blood glucose while HFD-fed female M-JAK2^−/−^ had lower random blood glucose levels than their M-JAK2^+/+^ counterparts (Fig. 3e). Moreover, male but not female M-JAK2^−/−^ mice fed HFD had a lower insulin resistance index (HOMA-IR) compared to their M-JAK2^+/+^ littermate controls (Fig. 3f,g). To further investigate the increase in systemic insulin sensitivity, we assessed AKT signaling in the VAT, liver and muscle upon insulin challenge in HFD-fed mice. We found that the insulin stimulation enhanced tyrosine phosphorylation of AKT at position serine 473 in comparison to non-insulin stimulated tissues (Fig. 3h-m). The enhancement of AKT phosphorylation was not different in VAT (Fig. 3h,i), liver (Fig. 3j,k) and muscle (Fig. 3l,m) of HFD fed M-JAK2^−/−^ mice compared with M-JAK2^+/+^ cohorts. Together, these observations show that M-JAK2^−/−^ mice demonstrate protection from HFD-induced systemic insulin resistance without differences in AKT signalling within liver, muscle and VAT.

### Macrophage JAK2 ablation attenuates HFD-induced inflammation in VAT and liver

**Figure 4:**
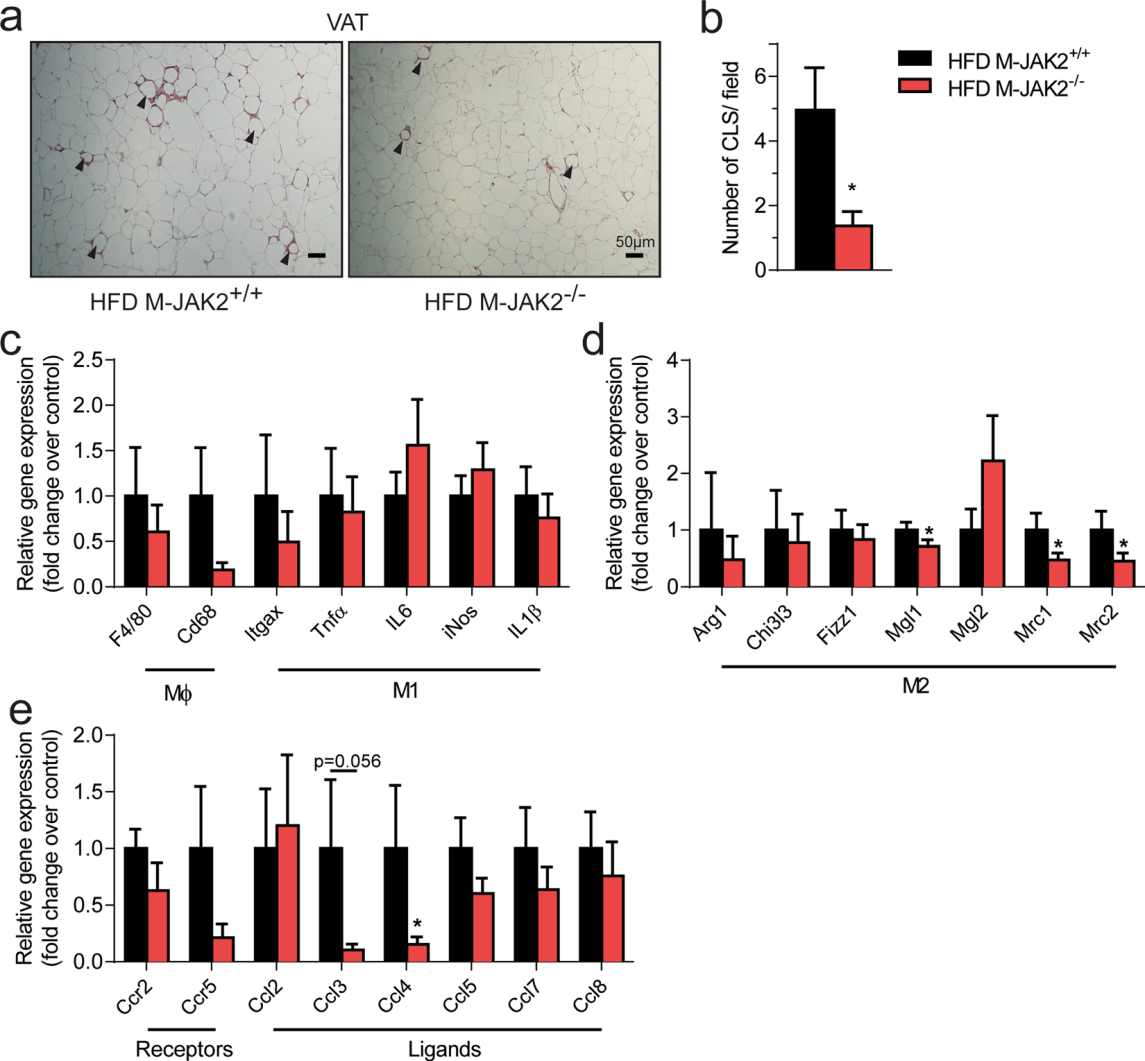
Decreased CLS and inflammation in VAT of HFD fed M-JAK2^−/−^ mice. (**a**) Representative micrographs of macrophage marker F4/80 stained VAT sections of HFD-fed M-JAK2^+/+^ and M-JAK2^−/−^ mice; crown-like structure (CLS) are indicated by arrowheads. (**b**) Quantification of CLS from VAT sections in at least 15 fields at 100X (n = 7–8). mRNA expression in VAT from HFD-fed M-JAK2^+/+^ and M-JAK2^−/−^ mice for (**c**) macrophage (Mϕ) and M1 markers, (**d**) M2 markers, and (**e**) chemokine receptors and its associated ligands (n = 8–11). All results are mean ± SEM; *p < 0.05.

Macrophage infiltration of white adipose tissue is implicated in the metabolic complications of obesity. A notable feature of adipose tissue in obese mice is the presence of unique clusters of macrophages that surround dead adipocytes called crown-like structures (CLS)^18^. To examine this, we immunostained VAT sections with macrophage marker F4/80 (Fig. 4a), which revealed a significant reduction in the presence of CLS in M-JAK2^−/−^ mice in comparison to M-JAK2^+/+^ mice after HFD feeding (Fig. 4b). These findings were supported by mRNA expression of inflammation and macrophage markers in VAT. There was a reduced expression of macrophage-specific gene *Cd68* (Fig. 4c) and reduction of some M2 markers, *Mgl1, Mrc1* and *Mrc2* (Fig. 4d), without significant differences in M1 markers. Since chemokines are another set of important cytokines implicated in tissue macrophage function, we evaluated the mRNA expression of chemokine receptor CC-motif chemokine receptor type 2 (CCR2) and its ligands (CCL2, CCL7, and CCL8) as well as CCR5 receptor and its ligands (CCL3, CCL4, CCL5, and CCL8). In VAT from HFD-fed M-JAK2^−/−^ mice, we observed a significant down-regulated expression for chemokine *Ccl4* with some others such as *Ccl3* and *Ccl5* along with expression of their associated receptor *Ccr5* that trended in the same direction (Fig. 4e). Together, our histological analysis and gene expression indicate that macrophage JAK2 deficient mice have dampened VAT inflammation.

**Figure 5:**
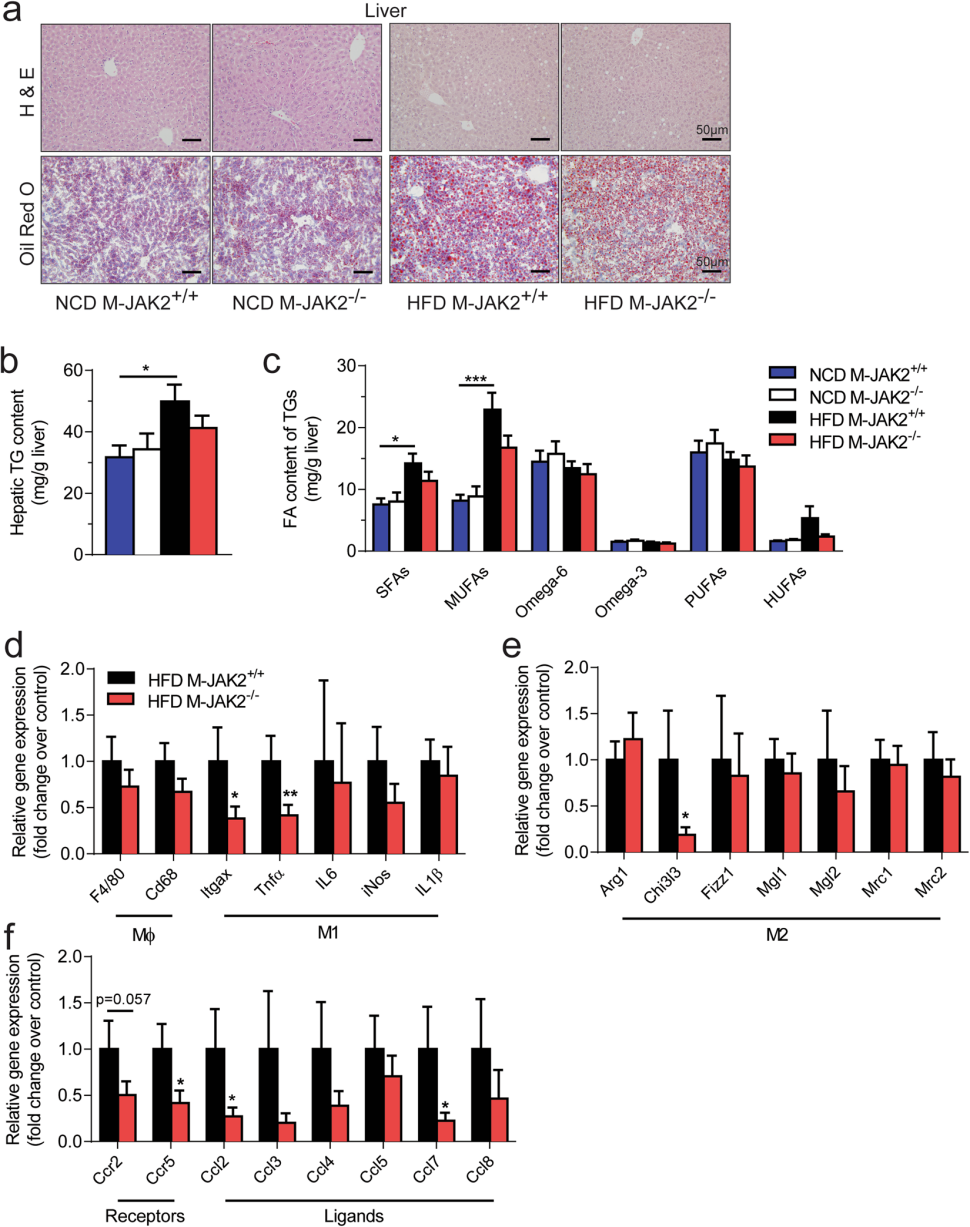
Reduced hepatic inflammation in HFD fed M-JAK2^−/−^ mice. (**a**) Representative micrographs of H&E (top) and Oil Red O (bottom) stained liver sections from NCD or HFD fed M-JAK2^+/+^ and M-JAK2^−/−^ mice. Quantification of (**b**) total triacylglycerol (TG) levels and (**c**) amount of saturated (SFAs), mono-unsaturated (MUFAs), Omega-6, Omega-3, polyunsaturated (PUFAs) and highly unsaturated (HUFAs) fatty acids (FA) of TGs in liver from NCD- (n = 4–6) and HFD- (n = 9–10) fed M-JAK2^+/+^ and M-JAK2^−/−^ male mice. mRNA expression in liver from HFD-fed M-JAK2^+/+^ and M-JAK2^−/−^ mice for (**d**) macrophage (Mϕ) and M1 markers, (**e**) M2 markers, and (**f**) chemokine receptors and its associated ligands (n = 8–9). All results are mean ± SEM; *p < 0.05, **p < 0.01, ***p < 0.001.

HFD induced obesity also leads to inflammatory changes in the liver, that is associated with insulin resistance and steatosis. We performed histological analysis on liver sections from NCD and HFD fed mice, and noticed increased lipid droplets in livers from HFD mice compared to NCD mice, as assessed by Oil Red O staining (Fig. 5a). Moreover, total hepatic triacylglycerol (TG) levels were increased with HFD feeding (Fig. 5b) along with saturated and monounsaturated fatty acids (Fig. 5c). Our analysis did not show any significant differences in TG levels or composition of fatty acids in TGs when we examined livers from macrophage JAK2 deficient mice compared to controls (Fig. 5b,c). To access the effect on hepatic inflammation with the lack of macrophage JAK2, we measured mRNA levels of inflammation and macrophage-related genes and found reduced expression of the cytokine *Tnfα* and macrophage markers *Itgax* and *Chi3l3* compared to controls (Fig. 5d,e). We also examined hepatic chemokine expression and observed decreased expression of *Ccr2, Ccr5*, *Ccl2* and *Ccl7* with macrophage JAK2-deficiency compared to controls (Fig. 5f).

**Figure 6:**
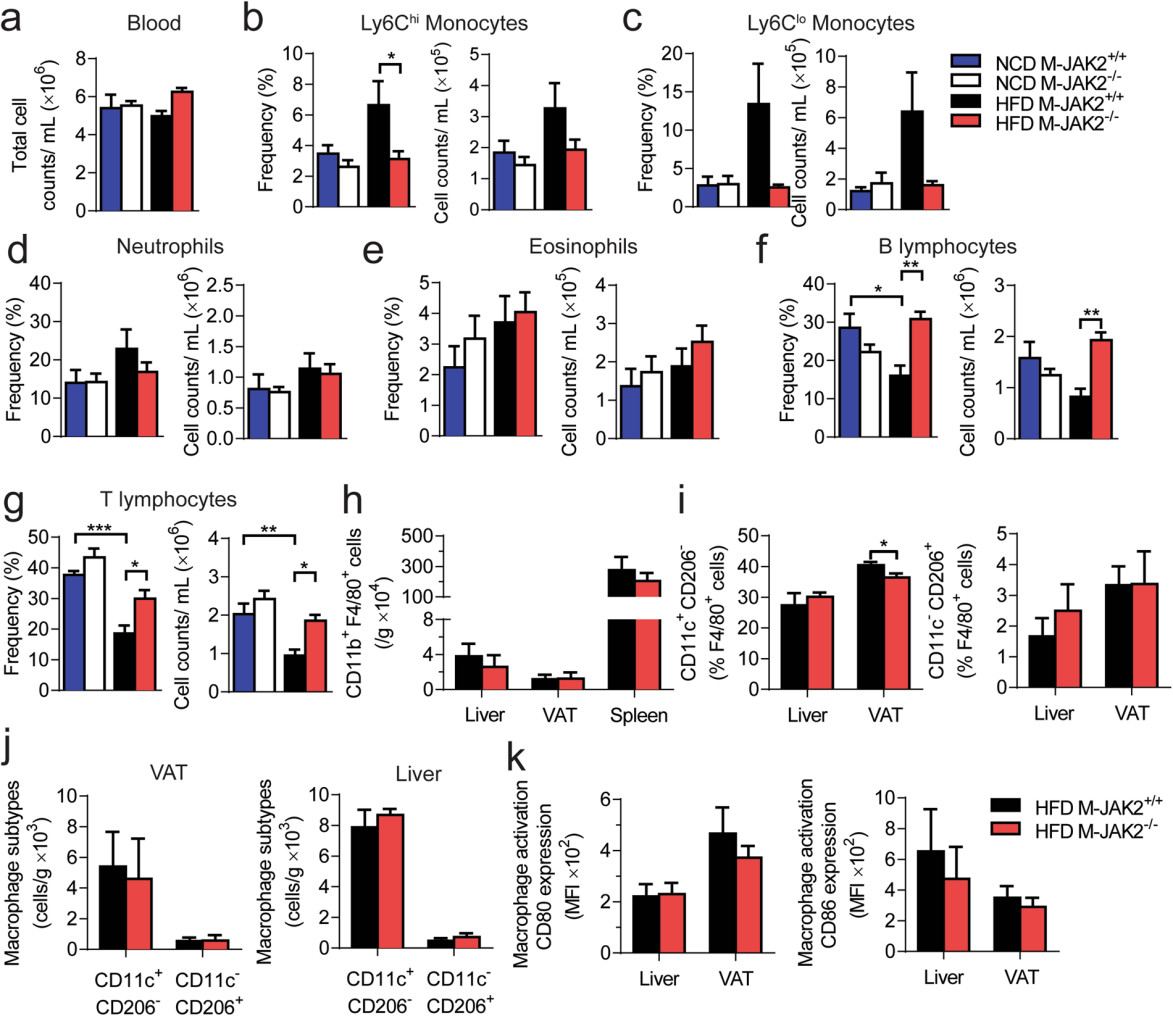
Characterization of circulating leukocytes, and macrophages in liver and VAT. (**a**) Total white blood cells, and (**b–g**) frequency and absolute number of (**b**) Ly6C high (Ly6C^hi^) monocytes, (**c**) Ly6C low (Ly6C^lo^) monocytes, (**d**) neutrophils, (**e**) eosinophils, (**f**) B lymphocytes, and (**g**) T lymphocytes in M-JAK2^+/+^ and M-JAK2^−/−^ mice fed a NCD (n = 5–7) or a HFD (n = 6–7). (**h**) Absolute numbers of CD45^+^CD11b^+^F4/80^+^ macrophages in liver, visceral adipose tissue (VAT) and spleen, (**i**) frequency and (**j**) absolute numbers of CD11c^+^CD206^−^ and CD11c^−^CD206^+^ macrophages in VAT and liver, and (**k**) expression of the macrophage activation markers CD80 and CD86 in the VAT and liver of M-JAK2^+/+^ and M-JAK2^−/−^ mice fed HFD (n = 8–9 mice per group for VAT and n = 5–6 per group for spleen and liver, two independent experiments). Mean fluorescent intensity (MFI). All results are mean ± SEM; *p < 0.05, **p < 0.01, ***p < 0.001.

Next, we measured various cytokines including adipokines and chemokines in circulation and did not find any differences except for an increase in IL-1β levels in HFD-fed M-JAK2^−/−^ compared to M-JAK2^+/+^ mice (Supplementary Fig. S2). There were no significant differences in circulating total white blood cells in HFD- or NCD-fed M-JAK2^−/−^ compared to controls (Fig. 6a). Interestingly, the increase in the number of circulating monocytes (Ly6C^hi^ and Ly6C^10^) seen with HFD feeding for M-JAK2^+/+^ mice was absent for M-JAK2^−/−^ mice (Fig. 6b,c). However, other myeloid cells such as neutrophils (Fig. 6d) and eosinophils (Fig. 6e) were not significantly different in numbers after NCD or HFD feeding. Furthermore, in accordance with previous reports noting the impact of obesity on lymphopoiesis^19, 20^, circulating B and T lymphocytes were decreased in numbers after HFD feeding compared to NCD fed controls (Fig. 6f,g). Interestingly, these changes in lymphocyte population did not occur in M-JAK2^−/−^ mice during HFD. Additionally, macrophage content (CD11b^+^F4/80^+^) as assessed by flow cytometry in liver and VAT, were not different in numbers in either of these tissues (Fig. 6h). Upon further analysis of these macrophages, we saw a decrease in frequency of CD11c^+^ CD206^−^ (M1) macrophages but no difference in frequency for CD11c^−^ CD206^+^ (M2) macrophages in HFD fed M-JAK2^−/−^ mice compared to controls (Fig. 6i,j). Additionally, expression of markers for macrophage activation (CD80 and CD86) in VAT and liver was also comparable between genotype groups (Fig. 6k). Taken together, these findings suggest that with HFD feeding macrophage JAK2 alters circulating leukocyte population and affects inflammatory changes at the tissue-specific level as demonstrated in liver and VAT.

### JAK2 regulates chemokine expression in macrophages

**Figure 7:**
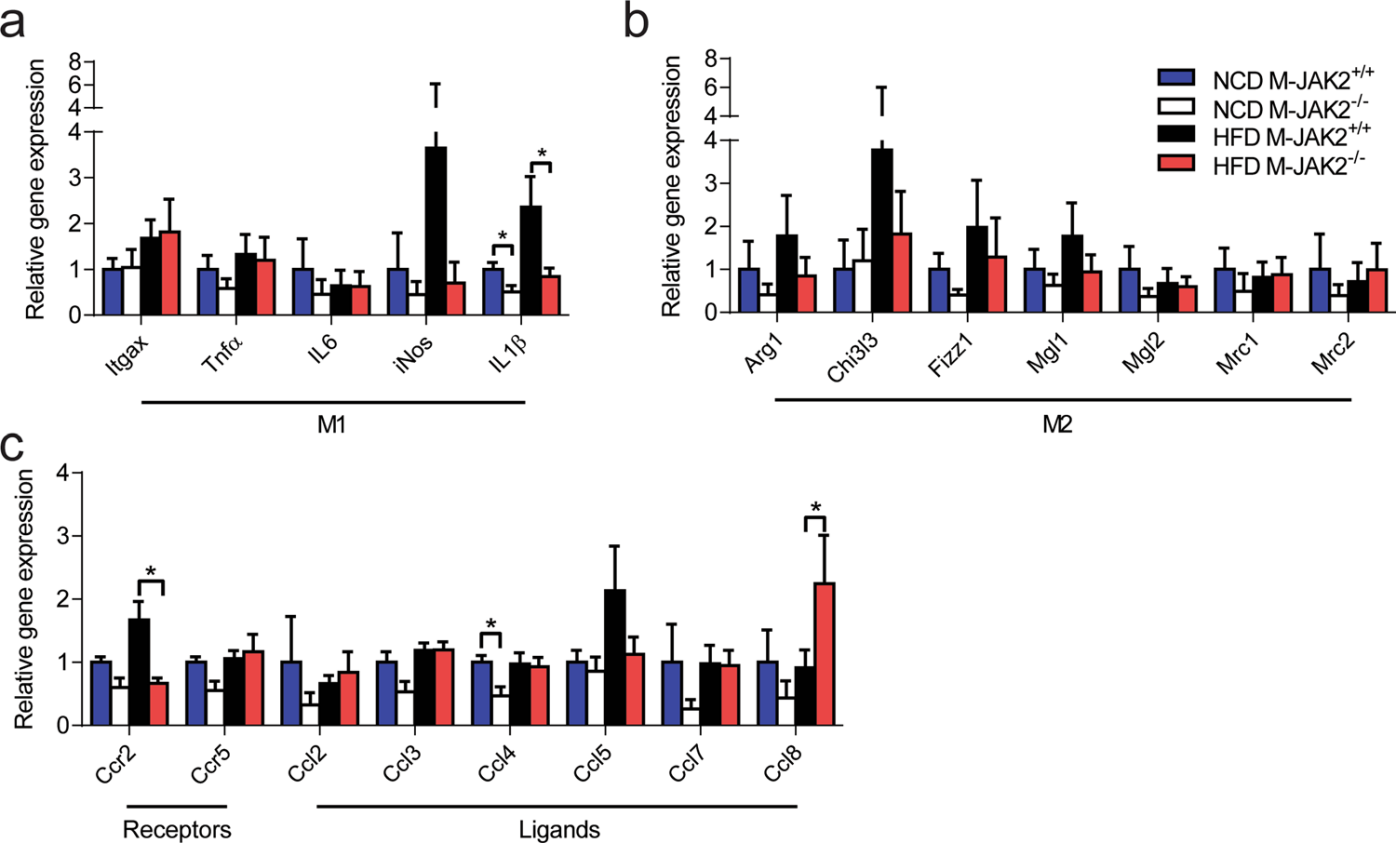
Cytokine measurements in peritoneal macrophages. mRNA expression as fold change over NCD-fed M-JAK2^+/+^ mice for (**a**) M1 markers, (**b**) M2 markers, and (**c**) chemokine receptors and its associated ligands in thioglycolate-elicited peritoneal macrophages from NCD (n = 3–4) or HFD (n = 5) fed M-JAK2^+/+^ and M-JAK2^−/−^ mice. All results are mean ± SEM; *p < 0.05.

To assess whether macrophages are the cells responsible for the suppressed HFD-induced chemokine expression in VAT and liver, we isolated PMs from both NCD- and HFD-fed M-JAK2^+/+^ and M-JAK2^−/−^ mice and examined the expression of chemokines and cytokines (Fig. 7a–c). PMs from NCD fed M-JAK2^−/−^ mice displayed significantly decreased expression of *Ccl4* and *IL1β* (Fig. 7a,c). On the other hand, PMs from HFD fed M-JAK2^−/−^ mice showed significantly reduced *Ccr2* and *IL1β* expression with an increased expression of *Ccl8* (Fig. 7a,c). M2 markers in PMs were not different between genotypes (Fig. 7b).

**Figure 8:**
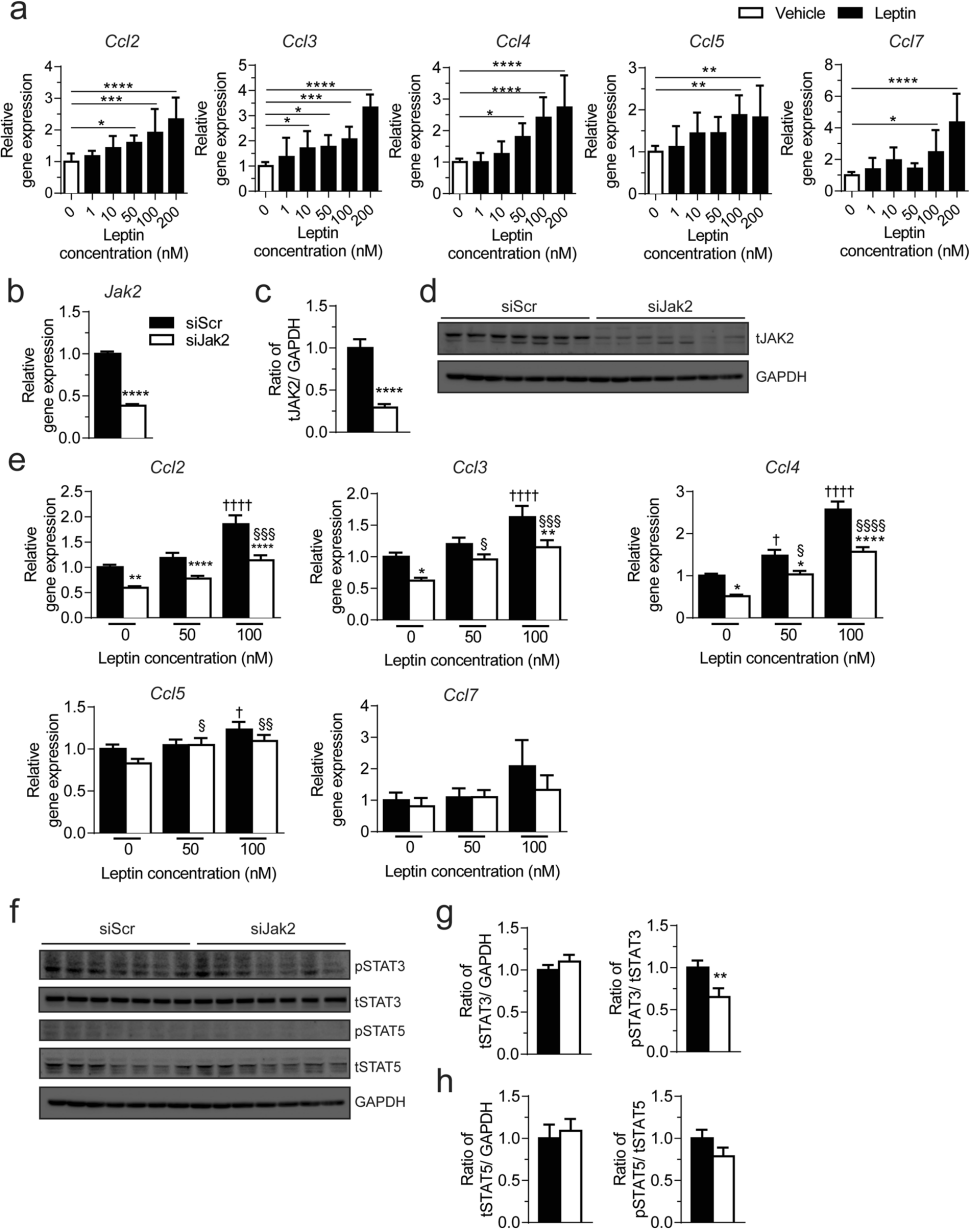
Chemokine expression in response to leptin in RAW 264.7 cells. (**a**) mRNA expression of chemokines as fold change over vehicle in macrophage cell line RAW 264.7 after stimulation with vehicle or varying concentrations of leptin. Three independent experiments performed in triplicates. (**b**) mRNA expression of *Jak2* in RAW 264.7 cells transfected with scramble siRNA control (siScr) or *Jak2* siRNA (siJak2) as fold change over siScr. (**c**) Quantification of total JAK2 levels relative to GAPDH, expressed as fold change over siScr, in RAW 264.7 cells transfected with siScr or siJak2 (n = 7). (**d**) Western blots of RAW 264.7 cells transfected with siScr or siJak2 for total JAK2 and GAPDH as a loading control. Full-length blots presented in Supplementary Fig. S9. (**e**) mRNA expression as fold change over vehicle-treated siScr of chemokines in response to vehicle or leptin (50 nM or 100 nM) in RAW 264.7 cells transfected with siScr or siJak2. Four independent experiments performed in triplicates; *siScr compared to siJak2, ^†^vehicle treated siScr compared to leptin-treated siScr, § vehicle-treated siJAK2 compared to leptin-treated siJak2. (**f**) Western blots for phospho-STAT3 (pSTAT3), total STAT3 (tSTAT3), phospho-STAT5 (pSTAT5), total STAT5 (tSTAT5), and GAPDH as a loading control in RAW 264.7 cells transfected with siScr or siJak2. Full blots in Supplementary Fig. S9. Quantification of protein levels as a fold change over siScr for (**g**) STAT3 and (**h**) STAT5 (n = 7). All results are mean ± SEM; *p < 0.05, **p < 0.01, ***p < 0.001, ****p < 0.0001.

We next sought to better understand the molecular mechanisms underlying reduced tissue inflammation with macrophage JAK2 deficiency, by first elucidating what could induce chemokine expression in macrophages. It has been shown that leptin, an adipokine primarily secreted by adipose tissue, can induce pro-inflammatory cytokine and chemokine production in macrophages^21^. Since leptin receptors are abundant on the surface of macrophages^22^ and JAK2 is known to be a main downstream mediator in leptin signaling^23^, we explored whether leptin might act to influence chemokine expression in macrophages. Indeed, upon leptin stimulation, expression of *Ccl2, Ccl3, Ccl4, Ccl5* and *Ccl7* were upregulated in a concentration-dependent manner (Fig. 8a). Subsequently, we assessed whether leptin signals through JAK2 to regulate chemokine expression in macrophages. We used a siRNA approach to effectively knockdown *Jak2* in macrophage cell line RAW 264.7 and we achieved over 60 percent reduction in *Jak2* mRNA expression (Fig. 7b) and about 70 percent reduction in JAK2 protein levels (Fig. 7c,d). Interestingly, *Jak2* knockdown significantly reduced mRNA levels of *Ccl2*, *Ccl3*, and *Ccl4* but had no effect on the expression of the chemokine receptors *Ccr2* and *Ccr5* or chemokines *Ccl5 and Ccl7* (Fig. 7e; Supplementary Fig. S3). Of note, mRNA levels of *Ccl2*, *Ccl3*, and *Ccl4* were reduced with *Jak2* knockdown even at basal conditions. Next, we evaluated chemokine expression in response to leptin and observed continued significant down-regulation in mRNA levels of *Ccl2*, *Ccl3*, and *Ccl4* in RAW 264.7 macrophages with *Jak2* knockdown (Fig. 7e). While chemokine expression in response to leptin was dampened in JAK2-deficient macrophages, their induction as measured by fold change in chemokine expression by comparing leptin to vehicle treatment was similar for RAW 264.7 cells transfected with scramble or *Jak2* siRNA (Supplementary Fig. S3).

In addition to JAK2’s role in inflammation, it also plays a role in lipid and cholesterol metabolism. Therefore, we sought to investigate whether expression of *Abca1*, *aP2, Cd36*, *Pparγ*, which are known to interact with or regulated by JAK2 signalling^12, 13, 24, 25^. The expression of these genes was not affected by *Jak2* knockdown in RAW 264.7 cells or *Jak2* deficient PMs (Supplementary Fig. S4). We also evaluated protein expression for targets downstream of JAK2 such as STAT3, STAT5, nuclear factor-kappaB p65 (NF-κB p65) and c-Jun NH2-terminal protein kinase (JNK) (Supplementary Fig. S5). Interestingly, total STAT3 levels were reduced in PMs from NCD fed M-JAK2^−/−^ mice while phosphorylation of STAT3 was lower for PMs from HFD fed M-JAK2^−/−^ mice compared to controls (Supplementary Fig. S5). Similarly, *Jak2* knockdown in RAW 264.7 cells reduced phosphorylation of STAT3 (Fig. 8f,h). The effect on protein expression of STAT5, NF-κB p65 and JNK were inconsistent in PMs or RAW 2647 cells lacking *Jak2* (Fig. 8f,h; Supplementary Fig. S5). Taken together, our *in vitro* work using RAW 264.7 cells shows that leptin upregulates chemokine expression in macrophages, which does not appear to be JAK2-dependent. In addition, knockdown of *Jak2* in RAW 264.7 cells or lack of *Jak2* in PMs diminishes chemokine expression and appears to disrupt STAT3 signaling.

## Discussion

The role of JAK2 during obesity and insulin resistance appears to be tissue-specific, as demonstrated by genetic ablation in the pancreas, liver and adipose tissue^12–16, 26^. In the liver, JAK2 has been shown to play a protective role in suppression of fatty liver and maintaining hepatic insulin sensitivity due to the importance of JAK2 in mediating hepatocellular growth hormone (GH) signalling^12, 13^. Similarly, JAK2 is important in regulating adipose tissue homeostasis with essential roles in the maintenance of insulin sensitivity^14, 15^ or activating thermogenesis during HFD or cold exposure^16^. Moreover, pancreatic JAK2 is important for erythropoietin signalling through this kinase with beneficial effects against diabetes development^26^; however, JAK2 does not appear to play a major role in regulating the compensatory increase in β-cell mass in response to HFD^27^.

It was previously not known whether macrophage JAK2 contributes to the development of obesity and insulin resistance. Various *in vitro* studies have provided insight into the dynamic function of macrophage JAK2 in mediating inflammatory response. Inhibition of JAK2 in murine macrophage cell lines by JAK2 specific inhibitor, AG490, suppresses the production of IL-6, IL-1β and iNOS upon phosphatidic acid or lipopolysaccharide stimulation^28, 29^. Several cytokines and hormones, for example, GH or GM-CSF signals through JAK2 to mediate expression of various cytokines and chemokines such as TNF-α, IL-1β, IL-12, and CCL2^30, 31^. In addition, a variety of other cytokines, including IFN-γ, IL-6, IL-3 and IL-13, have been demonstrated to signal through JAK2 to regulate macrophage function^32^. The diversity of factors that activate JAK2 within macrophages further implicates the importance of JAK2 signalling in these cells. Knowing that macrophages play a central role in orchestrating inflammatory responses and the involvement of JAK2 in regulating macrophage function, our work reveals that JAK2 deficiency in macrophage can have beneficial effects for reducing inflammation and improving insulin resistance induced by HFD. Indeed, our findings indicate that mice lacking JAK2 in macrophages had decreased mRNA levels of certain inflammatory and chemokine genes in PMs, and in VAT and liver after HFD feeding.

Macrophages within metabolic tissues can release pro-inflammatory cytokines and chemokines that act both locally and systemically to stimulate insulin resistance and the inflammatory response. The downregulation of cytokine and chemokine genes in tissues and macrophages, in addition to decreased CLS in VAT point towards a diminished inflammatory response with the loss of macrophage *Jak2*. Furthermore, in our macrophage *Jak2* knockout mice, we do not observe the decline in circulating lymphocytes that occur with HFD-feeding. These results suggest that macrophage JAK2 may have a role in regulating T and B lymphocytes, and this may also contribute to the regulation of VAT inflammation and CLS formation^33, 34^. The dampened inflammation in liver and VAT of macrophage *Jak2* knockout mice likely also contribute to the improvement of insulin sensitivity systemically. Despite the lack of difference in AKT signalling in the tissues examined, our data suggests a protective effect of macrophage *Jak2*-deficiency on HFD induced systemic insulin resistance along with reduced circulating glucose and insulin levels. The reduced VAT inflammation could also contribute to the reduced adipocyte hypertrophy and enhanced adipogenesis during diet-induced obesity in our macrophage *Jak2*-deficient mice. PPARγ and adiponectin are both important regulators of differentiation and renewal of adipocytes^35, 36^ and also play a role in maintaining adipose tissue and systemic insulin sensitivity^37, 38^. During inflammation, secreted factors derived from macrophages has also been shown to downregulate the expression of these adipogenesis genes^39, 40^.

Chemokines can also mediate the complex intracellular signalling that controls inflammation. We found that lack of macrophage JAK2 leads to lower expression of *Ccr2* and *Ccr5* and its associated ligands in liver and VAT. Interestingly, adipose tissue of obese subjects has elevated expression of these chemokines^41^. Moreover, deficiency of chemokine receptors, C*cr2* or C*cr5*, in mice leads to protection against HFD-induced adipose tissue macrophage accumulation^42, 43^. Previous studies have also suggested that CCR2 and CCR5, which belong to the superfamily of G-protein coupled receptors are capable of activating JAK2 in monocytes^44, 45^. In addition, various cytokines and growth factors, such as GM-CSF and leptin, can signal through JAK2 in macrophages to induce production of these chemokines^23, 31, 46^. Our work shows that macrophage *Jak2-*deficiency in PMs or RAW 264.7 cells had decreased expression of various chemokines and chemokine receptors, thus implicating the importance of macrophage JAK2 in mediating chemokine expression in liver and VAT.

The dampening of VAT inflammation in mice lacking *Jak2* also led us to investigate whether signalling of adipokine leptin through macrophage JAK2 may be the key modulator in the intercellular communication between adipocytes and adipose tissue macrophages. Leptin is considered a pro-inflammatory adipokine which can stimulate the production of inflammatory mediators such as cytokines (TNF-α, IL-6, IL-12)^21, 47^ as well as chemokines (CCL3-5, CXCL10)^21, 46, 48^ in monocytes and macrophages. It is also reported that incubation of human adipose tissue with chemokines such as CCL2 and CCL3 markedly decreased PPARγ protein expression while increasing leptin secretion by adipocytes^49^. We have shown that leptin induces expression of CC-chemokine ligands, CCL3, CCL4 and CCL5 as well as CCL2 and CCL7. However, this did not appear to be JAK2-dependent in macrophages. This could be explained by residual *Jak2* expression after the knockdown, or that leptin signals through non-canonical pathways such as mitogen-activated protein kinase (MAPK) and/or phosphatidylinositol 3-kinase (PI3K) to regulate chemokine expression after *Jak2* knockdown^23^.

Given the diversity of cytokines and growth factors signalling through JAK2, *Jak2* ablation may have either pro- or anti-inflammatory effects on macrophages. This may be due to the distinct tissue environments or the competing effects of cytokines which signal through JAK2 such as IFNγ and IL-13, which can activate M1 versus M2 phenotype, respectively. In our attempt to further identify mechanisms that might contribute to the downregulation of cytokines and chemokines in the absence of *Jak2* in macrophages, we observed that STAT3 phosphorylation is reduced in PMs and RAW 264.7 cells. Indeed, others have noted that STAT3 may be the predominant player downstream of JAK2 to regulate transcriptional activities of genes such as IL-1β^29^, iNOS^50^, and *Ccl3-5* ^46^ along with the importance of STAT3 activation downstream of CCR2^44^.

Overall, our study highlights the important role of macrophage JAK2 in peripheral metabolism. We show that genetic disruption of macrophage JAK2 reduces VAT and liver inflammation and improves systemic insulin sensitivity. Developing strategies to target macrophage JAK2 could potentially have a beneficial effect in attenuating insulin resistance and inflammation.

## Methods

### Animals

Macrophage *Jak2* knockout (M-JAK2^−/−^) mice were generated by breeding mice with the *Jak2* gene flanked by loxP sites^51^ with mice expressing the Cre recombinase under the control of the *LysM* promoter (Jackson Laboratory, Bar Harbor, ME, USA). The resulting *LyzM-Cre* ^+^ *Jak2* ^+*/ fl*^ mice were intercrossed to generate mice with or without *lox*P-flanked *Jak2* alleles and expressing the Cre recombinase, and the latter served as littermate controls (M-JAK2^+/+^). Mice were maintained on a mixed 129 Sv and C57BL/6 background, and housed in a pathogen-free facility at the Toronto Medical Discovery Tower (Toronto, ON, Canada) with a 12 h light–dark cycle and free access to water and standard irradiated rodent chow (5% energy from fat; Harlan Teklad). A cohort of mice was fed a HFD (60% fat, 24% carbohydrates and 16% protein based on caloric content; F3282; Bio-Serv) for 12 weeks starting at 6 weeks of age. All mice were maintained in a barrier facility at the Toronto General Research Institute and all animal experimental protocols were approved and performed in accordance with animal license guidelines and regulations established by the Toronto General Research Institute Animal Care Committee.

### Metabolic analyses

Body weight measurements were performed biweekly. All overnight fasts were 15 to 16 hours in duration. All blood glucose levels were determined from tail venous blood with an automated glucose monitor (One Touch II; Lifescan, Inc., Milpitas, CA). i.p. GTT was performed on overnight-fasted animals utilizing a glucose dose of 1.0 g/kg of body weight and i.p. ITT was performed on 4 hour-fasted animals using insulin lispro (B28Lys, B29Pro human insulin; Humalog, Eli Lilly) at a dose of 0.75 units per kg body weight. The homeostasis model assessment of insulin resistance (HOMA-IR) was calculated from the fasting blood glucose (mmol/L) × fasting plasma insulin (µU/ml) divided by 22.5. For insulin signaling experiments, mice fasted overnight were injected i.p. with insulin lispro (5 units/ kg). Tissues were removed 10 minutes later and snap-frozen. Real-time metabolic analyses were conducted in a Comprehensive Lab Animal Monitoring System (Columbus Instruments OH, USA). Mice were individually housed with free access to food and water to determine food intake, CO_2_ production, O_2_ consumption and activity counts for two consecutive light and dark cycles after at least 24 hours for acclimatization before data recording.

### Magnetic Resonance Imaging

MRI analysis was carried by the Spatio-Temporal Targeting and Amplification of Radiation Response (STTAR) program (Toronto, ON, Canada) in accordance with the Toronto General Research Institute Animal Care Protocol using a preclinical 7 Tesla system (Biospec 70/30, Bruker Corporation, Ettlingen), equipped with a B-GA12 gradient coil insert and 7.2 cm inner diameter linearly polarized RF volume coil. Mice, anaesthetized with 1.8% isoflurane, were oriented in prone position on a slider bed. Respiration was monitored using a pneumatic pillow (SA Instruments, Stony Brook, NY). Two-dimensional respiratory-gated RARE images were acquired as a series of 1 mm thick coronal sections encompassing the entire mouse (echo time = 32 ms, RARE factor = 8, repetition time determined by respiratory interval, matrix size = 360 × 160 providing 250 $\times$ 250 μm in-plane resolution over a 90 × 40 mm field-of-view, 8 averages, approximately 5.5 minutes scan time for a respiratory rate of 30 breaths-per-minute.

### Analyses of serum variables

Overnight-fasted mice were anesthetized and blood collected by cardiac puncture. Serum insulin levels were measured by a mouse insulin ELISA kit (Crystal Chem). Serum adipokines, cytokines and chemokines were measured using Milliplex Mouse Adipokine and Cytokine kit (Millipore).

### Histology analysis

Liver and perigonadal visceral adipose tissue (VAT) was removed, fixed and processed to paraffin blocks. Tissue sections were stained with haematoxylin and eosin (H & E). Adipocyte area and diameter were measured using H & E-stained VAT sections in 9 to 10 fields at 200 $\times$-power fields using cellSens software (Olympus, Tokyo, Japan). Adipocyte numbers were determined in 5 fields at 200 $\times$-power field. For Oil Red O staining, frozen liver sections were prepared using O.C.T. compound (Tissue-Tek) prior to staining (UHN Pathology Research Program, Toronto, ON, Canada).

Adipose tissue macrophages in VAT sections were detected by immunostaining for F4/80 (1:200 dilution, Santa Cruz Biotechnology) for 2 hours at room temperature (RT). Primary antibodies were detected using goat anti-rat IgG-biotinylated (1:200 dilution, Santa Cruz Biotechnology) with 30 minutes incubation at RT. Secondary antibodies were detected using streptavidin horseradish peroxidase (HRP) (Vector labs) for 30 minutes at RT followed by colour development Vector NovaRED Peroxidase (HRP) Substrate Kit (Vector labs) as per manufacturer’s instructions. CLS were quantified per 100 $\times$-power field in at least 15 fields in a blinded manner using Leica DM1000 microscope (Wetzlar, Germany) with Olympus DP72 camera (Waltham, MA, USA).

### Hepatic lipid analysis

Lipids were extracted from livers and TG content and fatty acid composition were quantified as previously described^12^.

### Isolation of peritoneal macrophages

Elicited peritoneal macrophages (PMs) were harvested 4 days after i.p. injection of 4% thioglycolate by lavage with ice-cold PBS containing 1% fetal bovine serum (FBS; Gibco). Cells were cultured overnight before collection for analysis.

### Cell culture

RAW 264.7 murine macrophage cells (TIB-71, American Type Culture Collection) were cultured as per manufacturer’s instruction. Briefly, PMs or RAW 264.7 cells were cultured in DMEM (4.5 g/L D-glucose) supplemented with 10% (vol/vol) FBS, 100 units/mL penicillin and streptomycin (Gibco) at 37 °C with 5% CO_2_ in humidified air.

### Leptin stimulation

RAW 264.7 cells were cultured in growth media supplemented with varying concentrations of recombinant murine leptin (450-31; PeproTech) for 6 hours before collecting cells for analysis. Three independent experiments were performed in triplicates.

### siRNA transfection

RAW 264.7 cells cultured in growth medium were transfected with 80 nM scramble siRNA or Silencer® Select *Jak2* siRNA (s68540, ThermoFischer Scientific) using Lipofectamine RNAiMAX reagent (Invitrogen) according to the manufacturer’s reverse transfection protocol. Cells were either collected 24 hours after transfection for analysis or treated with vehicle or leptin prior to collection. Four independent experiments were performed in triplicates.

### RNA isolation and quantitative RT-PCR

Total RNA from tissues, PMs or RAW 264.7 cells was isolated using Trizol reagent (Invitrogen). RNA was reverse-transcribed into cDNA and quantitative RT-PCR was performed using SYBR Green master mix on a 7900HT Fast-Real-Time PCR System (Applied Biosystems, Carlsbad, CA, USA). Each sample was run in triplicate in 10 μL volume. The relative mRNA abundance of each gene was normalised to the expression level of the housekeeping gene 18s.

### Western blotting

Tissues or cells were mechanically homogenized in ice-cold lysis buffer and centrifuged for 10 minutes at 4 °C and 14,000 *g* for tissues or 8,000 *g* for cells. The resulting supernatant fraction was separated by SDS-PAGE and immunoblotted with antibodies against phospho-AKT (S473; 1:1000), total AKT (1:1000), total JAK2 (1:1000), tubulin (1:1000), phospho-STAT3 (Y705, 1:2000), total STAT3 (1:1000), phospho-STAT5 (Y694, 1:1000), total STAT5 (1:1000), phospho-NF-κB p65 (Y694, 1:1000), total NF-κB p65 (1:1000), phospho-JNK (T183/Y185, 1:500, Santa Cruz Biotechnology) or total JNK1 (1:500, Santa Cruz Biotechnology) or GAPDH (1:5000). Antibodies were purchased from Cell Signaling Technology unless otherwise stated. Protein band intensity was quantified by ImageJ software.

### Flow cytometry

Immune cells were isolated from VAT and liver immune cells as previously described^52, 53^. To purify immune cells from spleen, we mechanically homogenized the spleen through a 70 μm nylon cell strainers followed by red blood cell (RBC) lysis and cell washing. Cells were resuspended in PBS containing 2% FBS and allowed to block nonspecific binding in Fc receptor blocking solution (BioLegend), followed by staining as previously described^53^ with the following fluorophore-conjugated primary antibodies: CD45.2 (104), CD11b (M1/70), Ly6G (1A8), CD11c (N418), CD206 (C068C2), F4/80 (BM8), CD80 (16-10A1), and CD86 (GL1) using recommended dilutions from the supplier (Biolegend).

For circulating leukocyte analysis, blood was collected from mice into heparin-coated capillary tubes and RBCs were lysed with RBC lysis buffer (Biolegemd). Cells were resuspended in FACS buffer and allowed to block nonspecific binding in Fc receptor blocking solution. Cells were incubated on ice for 30 minutes with fluorophore-conjugated primary antibodies: CD45 (EF-450), CD11b (M1/70), CD3e (145-2C11), B220 (30-F11), Ly6G (1A8), Ly6C (AL-21), and CD115 (AF598) using recommended dilutions from the supplier (BD Pharmigen). Data were acquired on a Fortessa or LSRII flow cytometer (BD Biosciences, San Jose, CA, USA) and analyzed with FlowJo software (Tree Star, Ashland, OR, USA).

### Statistical analysis

All data are presented as mean ± standard error of mean (SEM). Groups were compared with two-tailed Student’s t-test or multiple-group comparisons were performed using one-way ANOVA followed by Dunnett’s test or two-way analysis of variance (ANOVA) followed with Bonferroni’s test with GraphPad Prism (GraphPad Software, La Jolla, CA, USA). Comparison of energy expenditure as a function of body mass between genotype groups was analysed by ANCOVA. A value of *p* < 0.05 was considered statistically significant.

## Electronic supplementary material

Supplementary Figures S1-9
