# Supplementary material for: Macrophage JAK2 deficiency protects against high-fat diet-induced inflammation
Source: Sci Rep. 2017 Aug 9;7:7653. doi: 10.1038/s41598-017-07923-0 (PMC5550513; doi:10.1038/s41598-017-07923-0)
Supplement: Supplementary file 1 — Supplementary Figures S1-9 [file 41598_2017_7923_MOESM1_ESM.pdf]

# Macrophage JAK2 deficiency protects against high-fat diet-induced inflammation

Harsh R. Desai, Tharini Sivasubramaniyam, Xavier S. Revelo, Stephanie A. Schroer, Cynthia T. Luk, Prashanth R. Rikkala, Adam H. Metherel, David W. Dodington, Yoo Jin Park, Min Jeong Kim, Joshua A. Rapps, Rickvinder Besla, Clinton S. Robbins, Kay-Uwe Wagner, Richard P. Bazinet, Daniel A. Winer, Minna Woo

**Supplementary Figures**

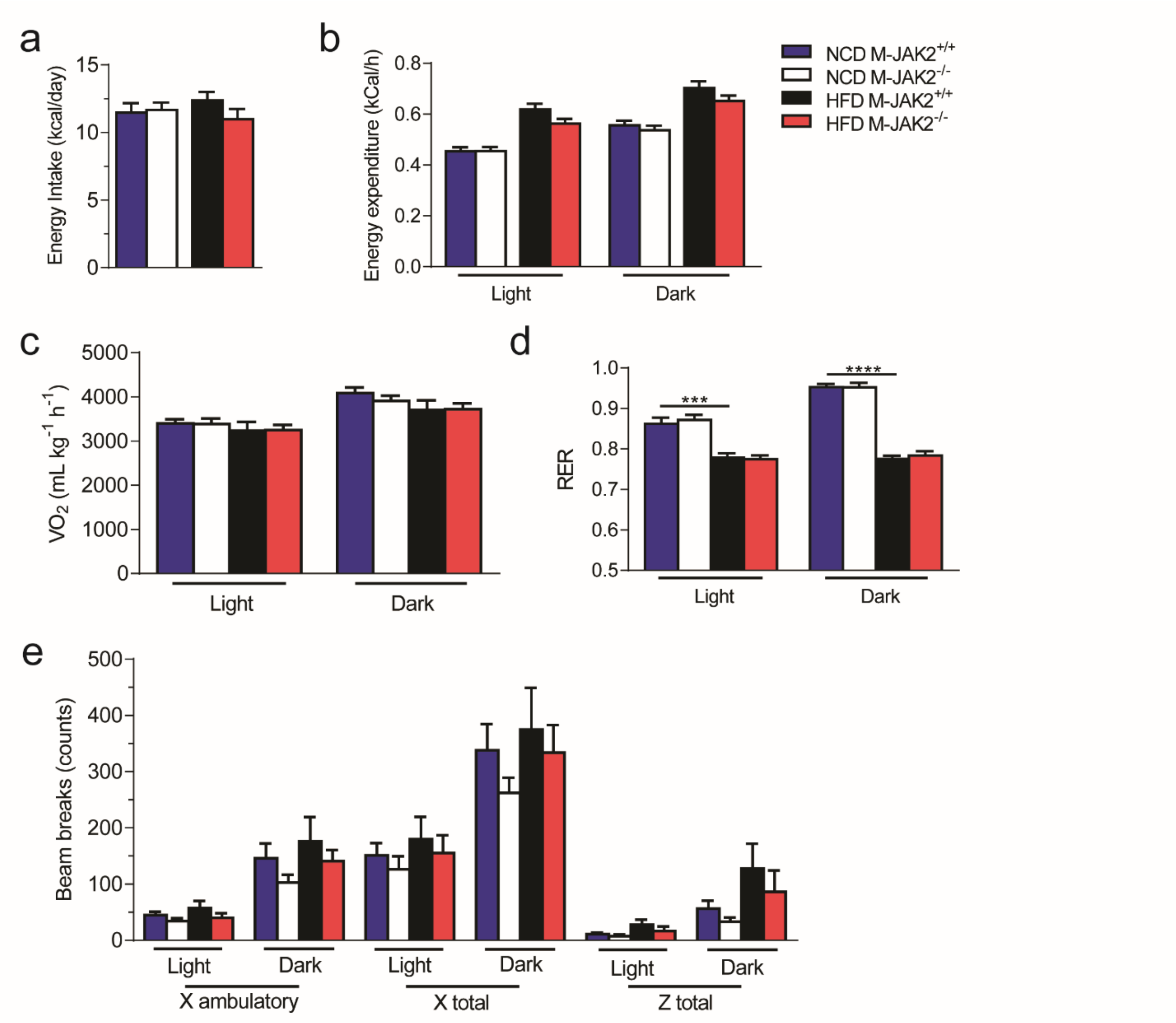


**Supplementary Figure S1: Metabolic measurements in NCD or HFD fed mice.**

Measurements of (a) energy intake, (b) energy expenditure analysed by ANCOVA with body weight as covariate; (c) oxygen consumption ($VO_2$), (d) respiratory exchange ratio (RER), and (e) activity level measured for M-JAK2$^{+/+}$ and M-JAK2$^{-/-}$ mice fed NCD (n= 6) or HFD (n= 5-7), respectively. All results are mean ± SEM; ***$p < 0.001$.

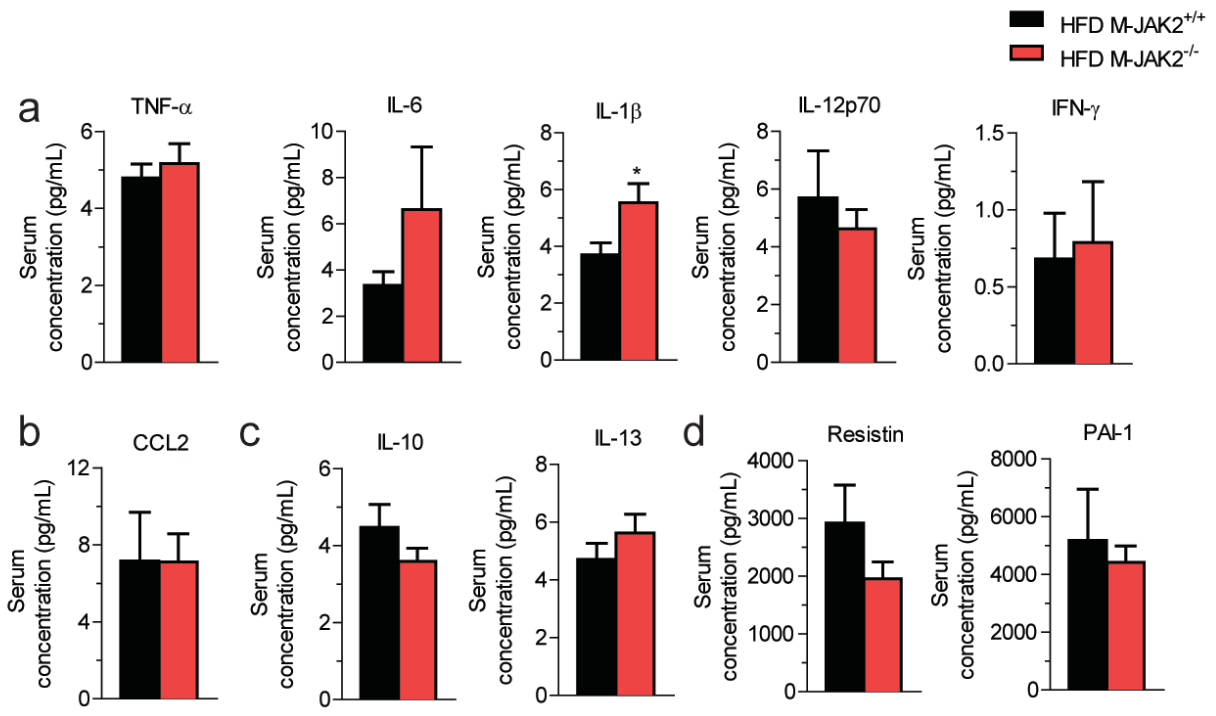


**Supplementary Figure S2**: **Assessment of circulating cytokines during HFD feeding.**

Fasting serum levels for (a,b) pro-inflammatory cytokines and chemokines (n= 8-11), (c) anti-inflammatory cytokines (n= 8-11) and (d) adipokines (n= 5) in for M-JAK2$^{+/+}$ and M-JAK2$^{-/-}$ mice fed HFD. All results are mean ± SEM; *p < 0.05.

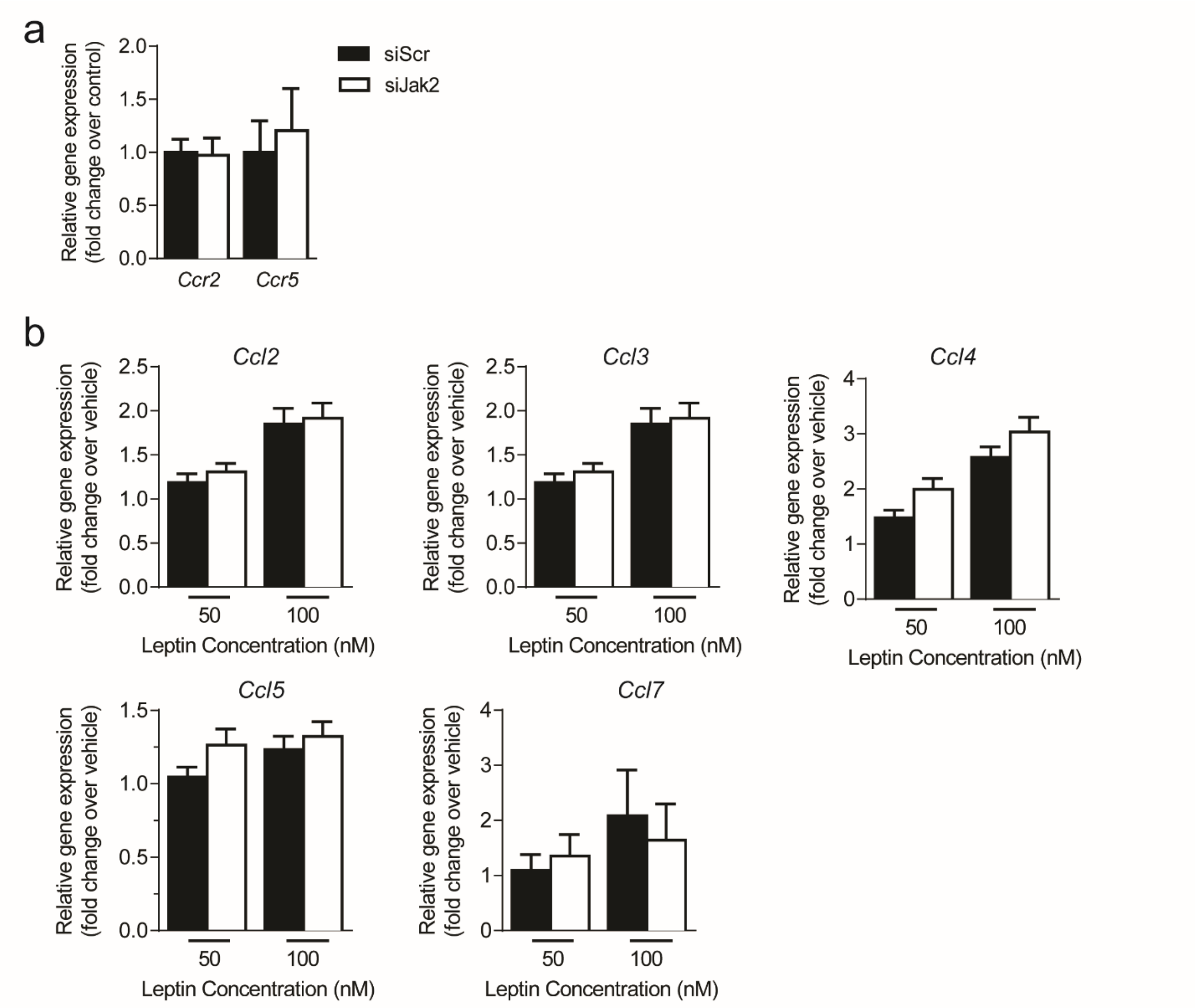


**Supplementary Figure S3: mRNA expression of chemokine receptors and fold change of leptin stimulated chemokine expression after Jak2 knockdown.**

(a) mRNA expression of chemokine receptors in macrophage cell line RAW 264.7 transfected with scramble siRNA control (siScr) or Jak2 siRNA (siJak2). (b) mRNA expression of chemokines after 50nM or 100nM leptin treatment expressed as a fold change of leptin treated compared to vehicle treated RAW 264.7 cells transfected with siScr or siJak2. Four independent experiments were performed in triplicates. All results are mean ± SEM.

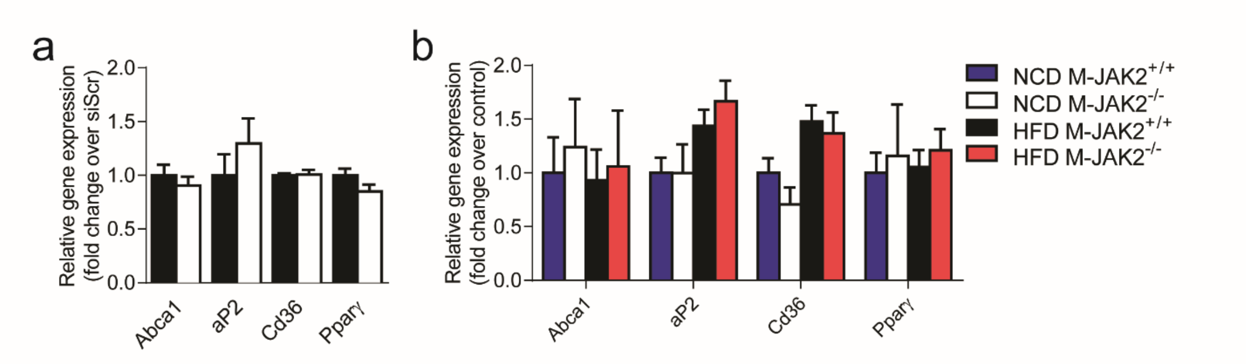


**Supplementary Figure S4: mRNA expression of genes involved in cholesterol and lipid metabolism in RAW 264.7 cells and peritoneal macrophages lacking JAK2**

mRNA expression of genes involved in cholesterol and lipid metabolism in (a) RAW 264.7 cells transfected with scramble siRNA control (siScr) or Jak2 siRNA (siJak2) and (d) peritoneal macrophages for M-JAK2$^{+/+}$ and M-JAK2$^{-/-}$ mice fed NCD (n= 3-4) or HFD (n= 5). Four independent experiments were performed in triplicates for RAW 264.7 cells. All results are mean ± SEM.

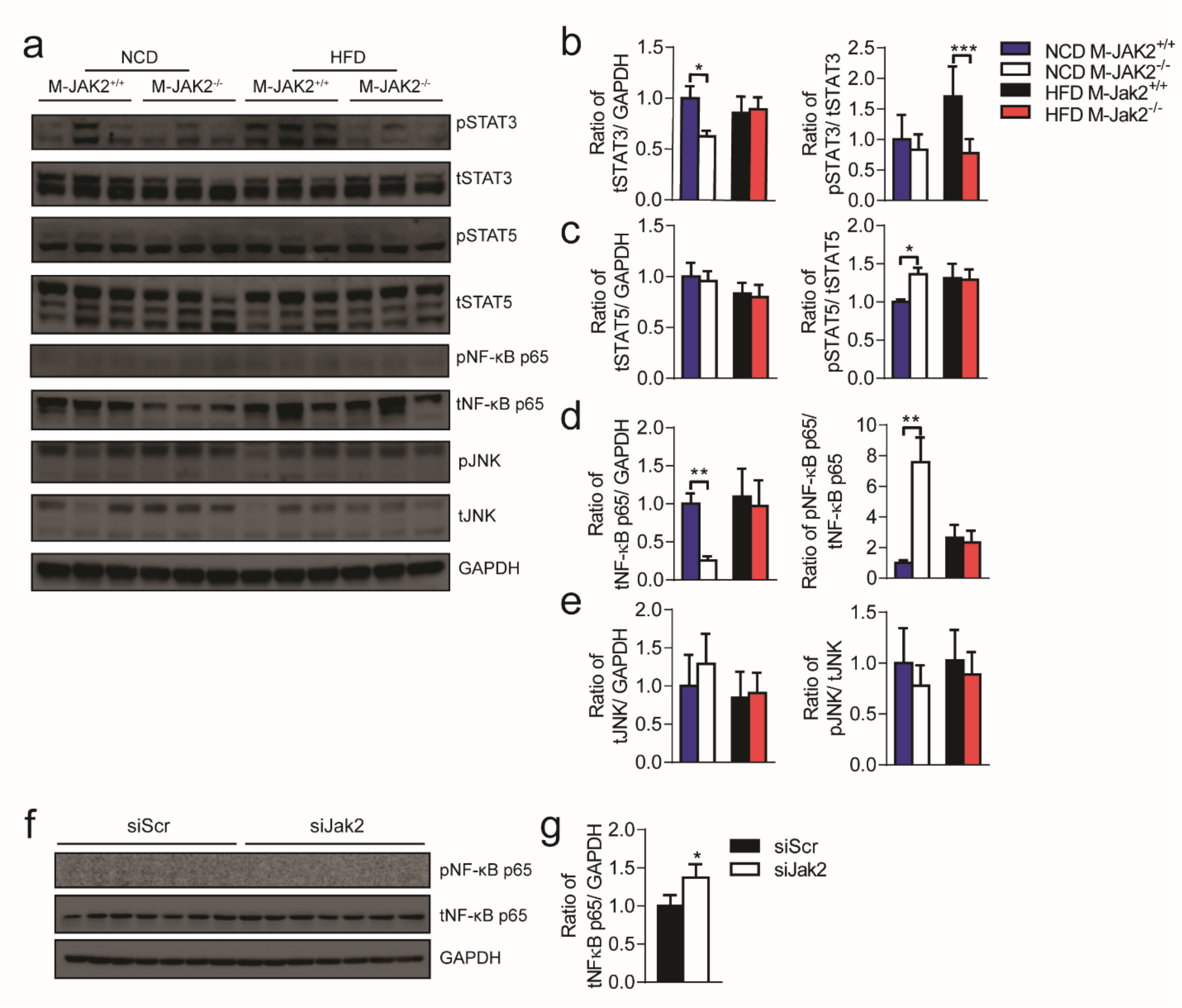


**Supplementary Figure S5: Protein expression analysis of certain factors downstream of JAK2 in peritoneal macrophages and RAW 264.7 cells**

(a) Western blots for phospho-STAT3 (pSTAT3), total STAT3 (tSTAT3), phospho-STAT5 (pSTAT5), total STAT5 (tSTAT5), phospho-NF-κB p65 (pNF-κB p65), total NF-κB p65 (tNF-κB p65), phospho-JNK (pJNK), total JNK (tJNK), and GAPDH (as a loading control) in peritoneal macrophages from NCD or HFD fed M-JAK2$^{+/+}$ and M-JAK2$^{-/-}$ mice. Full-length blots in Supplementary Fig. S8. Quantification of protein levels expressed as a fold change over NCD fed M-JAK2$^{+/+}$ for (b) STAT3, (c) STAT5 (d) NF-κB p65, and (e) JNK in peritoneal macrophages from NCD (n= 3) or HFD (n= 3) fed M-JAK2$^{+/+}$ and M-JAK2$^{-/-}$ mice. (f) Western

blots for phospho-NF-κB p65 (pNF-κB p65), total NF-κB p65 (tNF-κB p65), and GAPDH (as a loading control) in RAW 264.7 cells transfected with siScr or siJak2. Full-length blots are presented in Supplementary Fig. S9. (g) Quantification of protein levels expressed as a fold change over siScr for NF-κB p65 (n= 7). All results are mean ± SEM; *$p < 0.05$, **$p<0.01$, ***$p<0.001$.

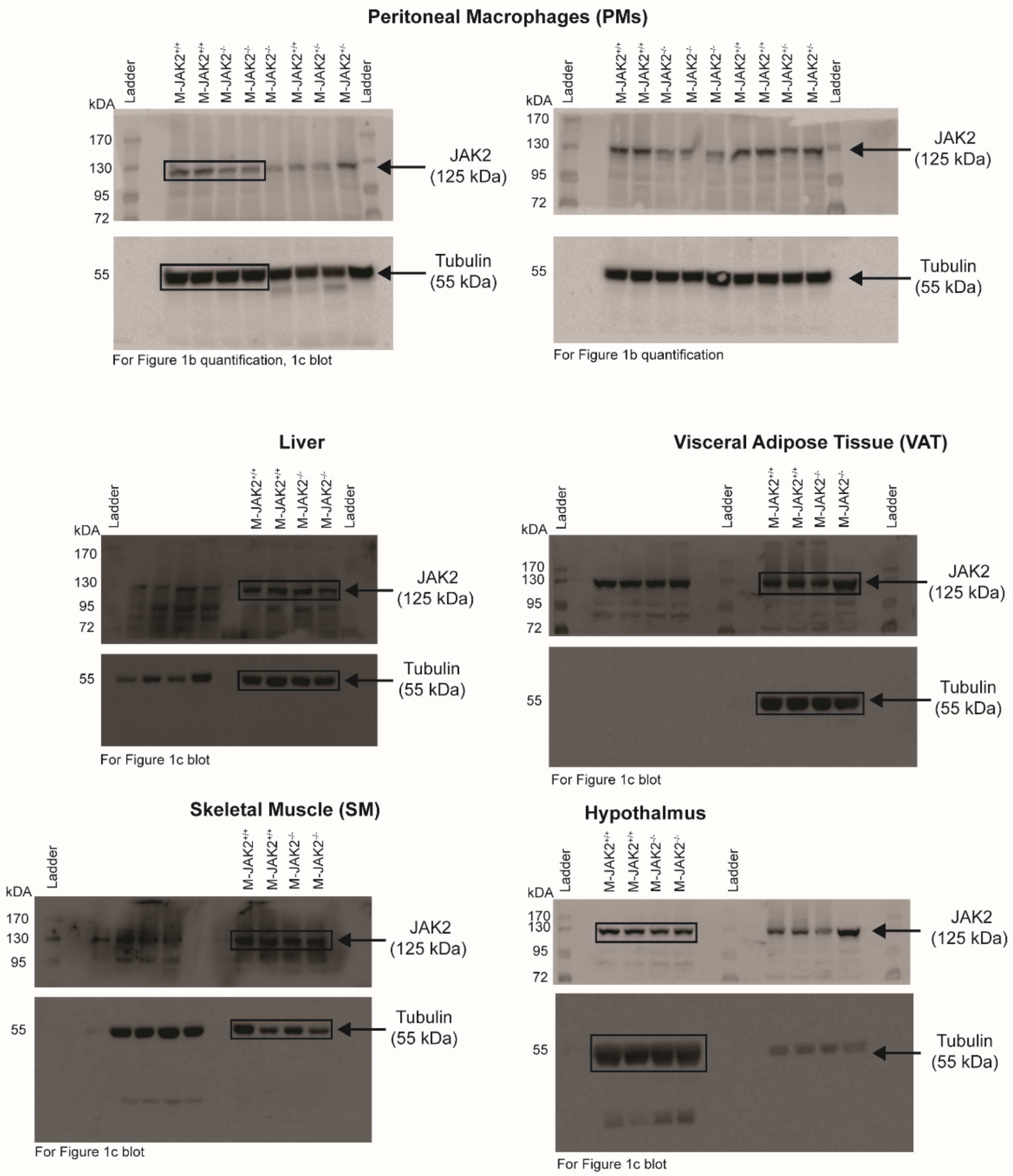


**Supplementary Figure S6: Uncropped full-length Western blots shown in Figure 1**

Original images of Western blots. Boxed areas indicate the cropped regions.

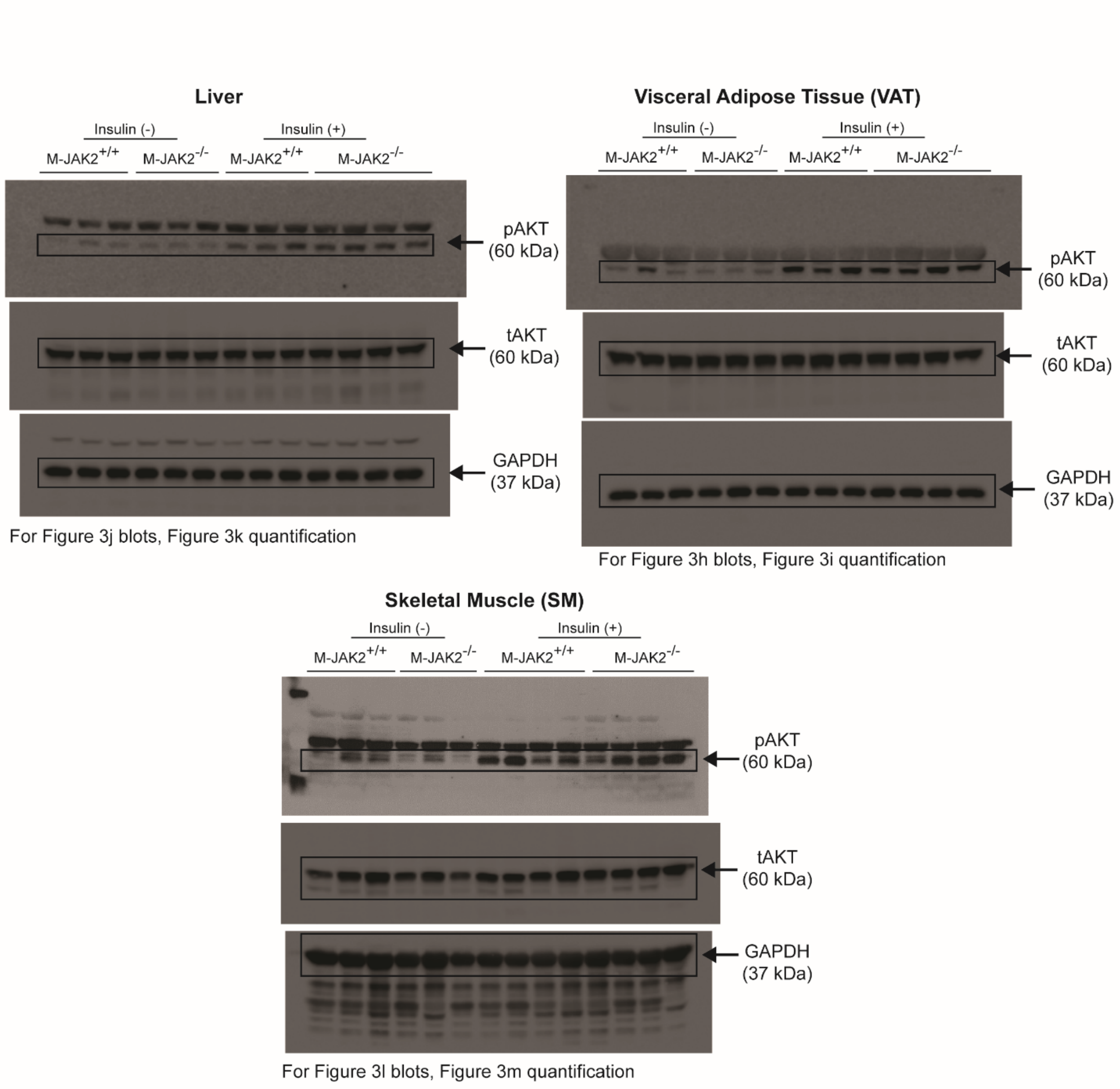


**Supplementary Figure S7: Uncropped full-length Western blots shown in Figure 3**

Original images of Western blots. Boxed areas indicate the cropped regions.

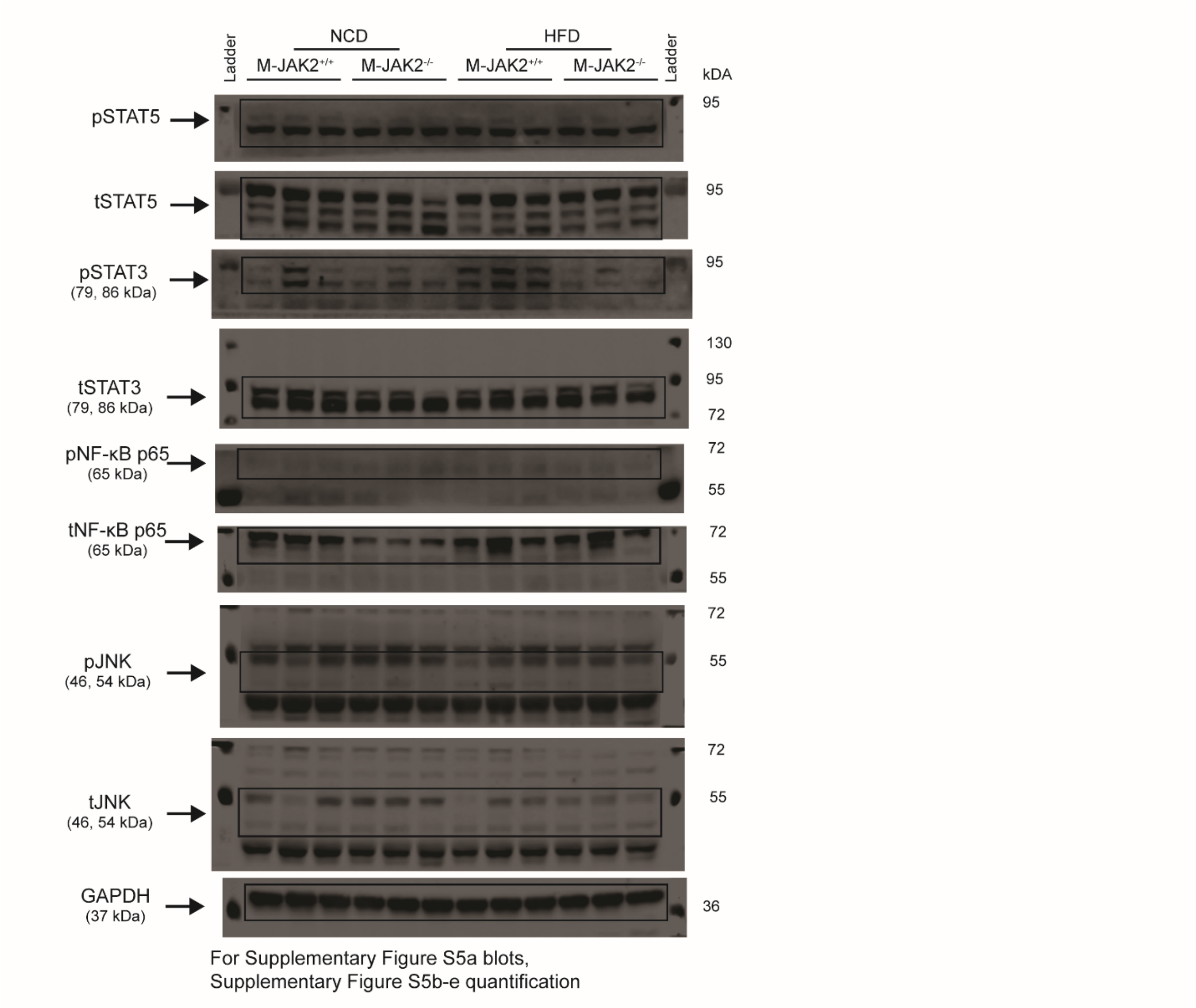


**Supplementary Figure S8: Uncropped full-length Western blots shown in Supplementary Fig. S5**

Original images of Western blots. Boxed areas indicate the cropped regions.

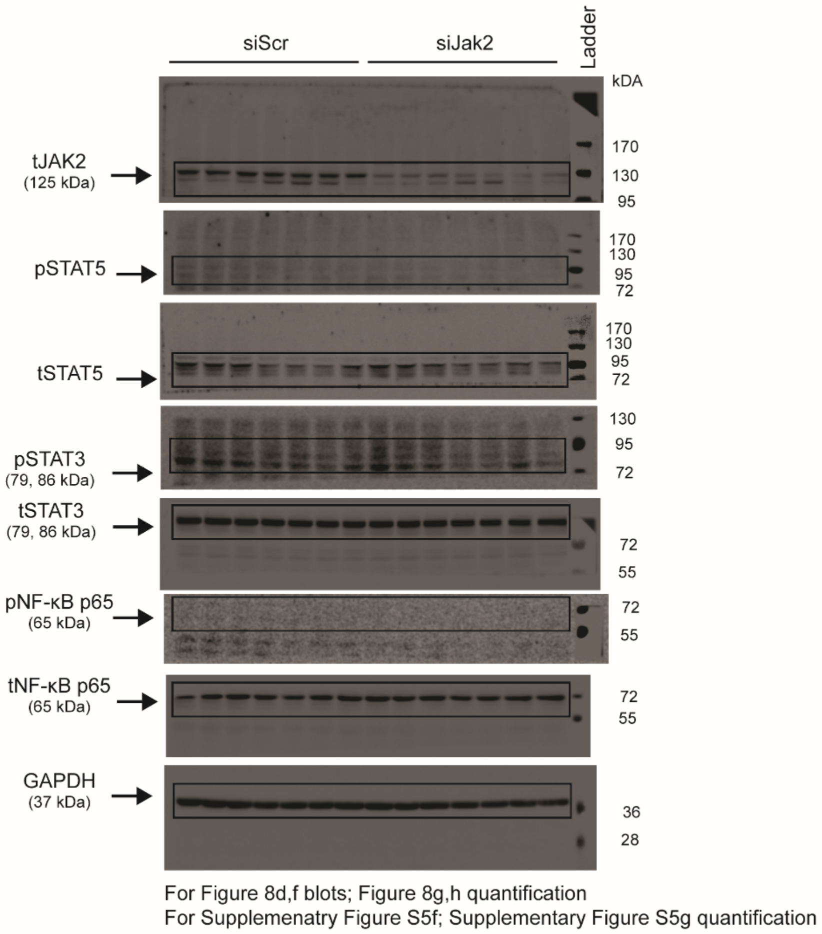


**Supplementary Figure S9: Uncropped full-length Western blots shown in Figure 8 and Supplementary Fig. S5**

Original images of Western blots. Boxed areas indicate the cropped regions.
